# Single‐cell RNA‐seq reveals clonal diversity and prognostic genes of relapsed multiple myeloma

**DOI:** 10.1002/ctm2.757

**Published:** 2022-03-16

**Authors:** Haiyan He, Zifeng Li, Jing Lu, Wanting Qiang, Sihan Jiang, Yaochen Xu, Weijun Fu, Xiaowen Zhai, Lin Zhou, Maoxiang Qian, Juan Du

**Affiliations:** ^1^ Department of Hematology Myeloma & Lymphoma Center Changzheng Hospital Naval Medical University Shanghai China; ^2^ Institute of Pediatrics and Department of Hematology and Oncology Children's Hospital of Fudan University National Children's Medical Center Shanghai China; ^3^ Shanghai Key Laboratory of Medical Epigenetics, International Co‐laboratory of Medical Epigenetics and Metabolism (Ministry of Science and Technology) Institutes of Biomedical Sciences Fudan University Shanghai China; ^4^ Department of Laboratory Medicine Changzheng Hospital Naval Medical University Shanghai China

**Keywords:** clonal diversity, multiple myeloma, relapse, single‐cell RNA‐seq, transcriptional features

## Abstract

**Background:**

Multiple myeloma (MM) is a clinically and biologically heterogeneous plasma‐cell malignancy. Despite extensive research, disease heterogeneity and relapse remain a big challenge in MM therapeutics. We tried to dissect this disease and identify novel biomarkers for patient stratification and treatment outcome prediction by applying single‐cell technology.

**Methods:**

We performed single‐cell RNA sequencing (scRNA‐seq) and variable‐diversity‐joining regions‐targeted sequencing (scVDJ‐seq) concurrently on bone marrow samples from a cohort of 18 patients with newly diagnosed MM (NDMM; *n* = 12) or refractory/relapsed MM (RRMM; *n* = 6). We analysed the malignant clonotypes using scVDJ‐seq data and conducted data integration and cell‐type annotation through the CCA algorithm based on gene expression profiling. Furthermore, we identified disease status‐specific genes and modules by comparison of NDMM and RRMM datasets and explored the findings in a larger MM cohort from the MMRF CoMMpass study.

**Results:**

We found that all the myeloma cells in either diagnosed or relapsed samples were dominated by a major clone, with a few subclones in several samples (*n* = 5). Next, we investigated the universal transcriptional features of myeloma cells and identified eight meta‐programs correlated with this disease, especially meta‐programs 1 and 8 (M1 and M8), which were the most significant and related to cell cycle and stress response, respectively. Furthermore, we classified the malignant plasma cells into eight clusters and found that the cell numbers in clusters 2/6/7 were exclusively higher in relapsed samples. Besides, we identified several attractive candidates for biomarkers (e.g. *SMAD1* and *STMN1*) associated with disease progression and relapse in our dataset and related to overall survival in the CoMMpass dataset.

**Conclusions:**

Our data provide insights into the heterogeneity of MM as well as highlight the relevance of intra‐tumour heterogeneity and discover novel biomarkers that might be a potent therapy.

## Graphical abstract

Single‐cell RNA sequencing and variable‐diversity‐joining regions‐targeted sequencing revealed clonal diversity. The universal transcriptional features of myeloma cells were investigated, and eight meta‐programs correlated with this disease were identified. Several attractive candidates for biomarkers (e.g., SMAD1 and STMN1) associated with disease progression were identified, which were validated by functional investigation and confirmed overall survival related in the CoMMpass dataset.

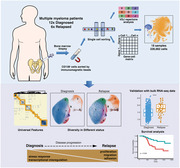

## 1 INTRODUCTION

Multiple myeloma (MM) is an incurable plasma cell disorder.^1^ Advances in anti‐myeloma therapeutics during the last decades have considerably improved the treatment outcome in myeloma.^2^, ^3^ Despite these improvements, MM remains incurable, with almost all patients unavoidably relapsing and dying from this disease.^4^ Several valuable genetic biomarkers for disease progression and relapse have been identified in previous studies using the methodology of karyotyping, fluorescence in situ hybridization (FISH) and targeted sequencing,^5^ and results from large‐scale whole‐genome sequencing studies^6^, ^7^, ^8^, ^9^ have revealed the genetic landscape of this disease. However, these technologies lack depth and resolution for defining the complicated cellular composition and pathways driving MM disease progression and relapse. It is essential to develop new tools for risk stratification and accurate patient therapeutic response prediction because of the progressive and continually evolving nature of the malignant cells, which may substantially improve therapeutic management. Thus, novel molecular biomarkers for more successful patient stratification and treatment response prediction are needed. Single‐cell sequencing technology opens a way for characterizing the tumour cells and disease progression in an unprecedented resolution.^10^ Applying this technology, several studies have already identified a series of significant genes or modules, which correlated with plasma cell heterogeneity,^11^ disease pathogenesis,^12^ immune microenvironment,^13^, ^14^ resistance pathways and therapeutic response^15^, ^16^ in MM. In this study, we performed combined single‐cell RNA sequencing (scRNA‐seq) and scVDJ‐seq using the 10× Genomics system to analyse the transcriptional profiles and clonotypes concurrently for 12 newly diagnosis MM (NDMM) and 6 relapsed/refractory MM (RRMM) samples. Our results indicated that all the myeloma cells in either diagnosis or relapse samples were dominated by a major clone, with a few subclones in five samples. Furthermore, we investigated their universal transcriptional characteristics, clustered the malignant plasma cells and identified several potential biomarkers (e.g. *SMAD1* and *STMN1*) associated with disease progression and relapse in our dataset and related to overall survival (OS) in the CoMMpass dataset.

## 2 MATERIALS AND METHODS

### 2.1 Samples collection

Patient samples were acquired with patients’ written consent in accordance with the Declaration of Helsinki with approval from the ethics committee of Shanghai Changzheng Hospital. Bone marrow (BM) samples were acquired from 12 NDMM and 6 RRMM. Plasma cells were sorted using CD138‐coated magnetic beads (MACS; Miltenyi Biotec). Tumour cell purity was estimated using a slide‐based assay and/or flow cytometry (Figure S1).^17^

### 2.2 Single‐cell 5′ mRNA and VDJ sequencing

After MACS selection, all CD138^+^ cells were encapsulating separated into droplets, and libraries were constructed using the Single Cell 5' Library & Gel Bead Kit (10x Genomics) Chromium and Single Cell V(D)J Enrichment Kit for B cells, following the manufacturer's instructions. The libraries were finally sequenced using an Illumina Novaseq 6000.

### 2.3 ScRNA‐seq data processing and quality control

Raw sequencing data were converted into FASTQs using the Illumina's bcl2fastq software. FASTQ files were aligned to the human genome (GRCh38) using the *CellRanger* (version 3.1.0) pipeline. The initial gene expression matrix was then processed and analysed by *Seurat* package (version 3.1.5)^18^ unless otherwise stated. We first used *Scrublet* ^19^ to exclude doublets by predicting potential cell doublets. Then, the cells were included with genes greater than 500, the counts of RNA per cell greater than 2000, the percentage of mitochondrial reads less than 10% and the percentage of haemoglobin reads less than 2%. The basic information for single‐cell datasets of samples can be seen in Table S1.

### 2.4 Dimensionality reduction, clustering and visualization

After filtering, the expression matrix was normalized by the *NormalizeData* function in the *Seurat* package and ln‐ transformed [ln (CPM+1)]. Then, principal component analysis was applied with the highly variable genes as input using ‘*RunPCA*’. Clustering was applied with graph‐based clustering algorithm and visualized with Seurat functions ‘*RunTSNE*’ and ‘*RunUMAP*’.

### 2.5 Cell type's identification

Cell types were assigned by examining the mean expression of classical markers manually. The markers used were *PTPRC* (immunocytes), *CD19, MS4A1, CD79A* (B cells), *CD3, CD4, CD8* (T cells), *CD14, FCGR3A, LYZ* (monocytes) and *SDC1* (myeloma cells) according to the literature.^20^, ^21^

### 2.6 scVDJ analysis

Contig assembly, annotation and clonotype analysis were performed using ‘*cellranger vdj*’ with the Cell Ranger V(D)J compatible reference: (refdata‐cellranger‐vdj‐GRCh38‐alts‐ensembl‐3.1.0). Assembled contigs labelled as low‐confidence, non‐productive or with UMIs < 2 were discarded. To identify the BCR clonotype for each malignant cell, only cells with at least one heavy chain (IGH) and one light chain (IGL or IGK) were kept. For a given malignant cell, if two or more IGH or IGL/IGK assembled, the highest expression level (UMI or reads) IGH or IGL/IGK was defined as the dominated IGH or IGL/IGK in the cell. Each unique dominated IGH‐IGL/IGK pair (CDR3 nucleotide sequences and rearranged VDJ genes) was defined as a clonotype. Malignant cells with the same clonotype constituted a tumour clone. We defined the tumour clone that constitutes > 75% of malignant cells with defined clonotypes as the major clone, with .1‐10% of malignant cells as minor clones. All the other tumour clones were identified as intermediate clones.

### 2.7 inferCNV analysis

The copy number variation (CNV) analysis based on the filtered expression matrix was conducted by *infercnv* R package (version 1.2.1). For this analysis, the default parameters for 10x Genomics data were used. We extracted feature vectors regarding chromosome 1q gain, 13q loss and 17p loss status from a table ‘map_metadata_from_infercnv.txt’ to show the CNV signatures of MM, which were demonstrated before.

### 2.8 Deciphering intra‐tumour expression programs and meta‐programs of MM

The consensus non‐negative matrix factorization (cNMF) algorithm^22^ was used for the myeloma cells of each sample individually. *NMF* was run 200 times for components (*k*) from 2 to 25 signatures. For each *k*, the 200 repetitions are clustered in *k* groups. To determine the number of components (*k*), we used the diagnostic plot according to the tutorial (https://github.com/dylkot/cNMF). We obtained between 10 and 19 intra‐tumour expression programs per sample, 234 programs in total. From the intra‐tumour expression program Pearson correlations, we used hierarchical clustering to find trends of programs. Eight main meta‐programs emerged.

### 2.9 Meta‐program score calculation

To avoid bias of random selection of genes in the meta‐program definition, we ranked the marker genes of each intra‐tumour expression program by the frequency of the occurrence in each meta‐program. The top 30 genes were selected, and the genes which appeared in less than three intra‐tumour immune programs were excluded. The ‘*AddModuleScore*’ function in *Seurat* was applied for calculating the meta‐program score for each single‐cell sample.^23^, ^24^

### 2.10 Integrated analysis based on myeloma cells

To handle batch effects, we utilized the canonical correlation analysis (*CCA*)^25^ in the *Seurat* package for data integration. For this analysis, the following parameters were used: default top 2000 genes with the highest dispersion from 18 datasets, and the first 30 canonical correlation vectors.

### 2.11 Differential gene expression analysis

To find markers that define cell clusters, we performed differential expression analysis on data using *Pegasus* (version 1.0.0) (Mann‐Whitney U test, log2FC and AUROC are calculated). In the context of the differential gene expression analysis, the selected genes for downstream analysis utilized a default threshold of .5 for average log2FC.

### 2.12 SCENIC analysis

The SCENIC analysis was applied by *pySCENIC* package (version 0.10.3), following the protocol on https://github.com/aertslab/SCENICprotocol.

### 2.13 RNA velocity analysis

The RNA velocity analysis was applied by *scVelo* package (version 0.2.2), following the protocol on https://scvelo.readthedocs.io.

### 2.14 Processing of bulk RNA‐seq data and survival analysis

The bulk RNA‐seq raw data of BM samples combined with clinical data from 843 samples, including NDMM (*n* = 763) and RRMM (*n* = 80), were downloaded from the TCGA (https://tcga‐data.nci.nih.gov/) MM RNA sequencing dataset (MMRF‐CoMMpass). Patients without BM samples or complete survival information were excluded. The module score was calculated by dividing the FPKM value of each meta program in each sample, followed by log2 transformation. Survival times were plotted using Kaplan‐Meier survival curves and analysed using log‐rank tests.

### 2.15 Reverse transcription‐quantitative polymerase chain reaction assay for RNA transcript expression

Total RNA was isolated from cells using Trizol reagent (TIANGEN Biotech, Beijing). Five hundred nanograms of purified RNA was reverse transcribed using the Hifair 1st Strand cDNA Synthesis SuperMix for qPCR (gDNA digester plus) (Y Cat. ID:KR116, TIANGEN Biotech). PCR samples were prepared with diluted cDNA (1:30), 5 μl SYBR Green PCR master mix (TIANGEN Biotech), .2 μM each of the forward and reverse primers (Table S2) in a total volume of 10 μl. Reverse transcription‐quantitative polymerase chain reaction (RT‐qPCR) was performed by using an Analytikjena qPCRsoft 4.0 (Germany). The relative expression level of the target gene was calculated by using the 2−ΔΔCT method and graphed as fold change (2−ΔΔCT) from control.

### 2.16 Gene silencing and siRNA transfection

The myeloma cell lines NCI‐H929, RPMI8226 and U266 were cultured according to the protocol. The transient siRNA and the transfection reagent INTERFERin® (polyplus corporation) were used in this study. The siRNA sequences are listed in Table S3 with the concentration at 15 nM. After transfection, the cells were cultured in a 5% CO_2_ incubator at 37°C for 48 h, and the samples were collected for detection.

### 2.17 Detection of cell viability by CCK8

Three myeloma cell lines were plated in 96‐well plates, 2.0×10^4^ cells per well and transfected according to the above transfection method. After 48 h of culture, 1/10 volume of WST‐8 solution was added to the culture medium for 2 h. The OD value was measured by a microplate reader. The Enhanced Cell Counting Kit‐8 used in this experiment was purchased from Beyotime Biotechnology Co., Ltd., China (Cat. ID: C0042).

### 2.18 Apoptosis and cell cycle assays

Cells were transfected with siRNA and cultured for 48 h. After staining with annexin V‐FITC/PI (Beyotime Biotechnology Co., Ltd., Cat. ID: C1062), the results were detected by flow cytometry and analysed by FolwJo software. For cell cycle assay (reagents from Beyotime Biotechnology Co., Ltd., Cat. ID: C1062), the cells were fixed with 70% ethanol, stained with PI and detected by flow cytometry after 48 h culture.

### 2.19 Statistical analysis

Statistical analysis was performed in R (version 4.1.0) or Python (3.7.10). All results in graphs are presented as means ± s.d. unless specified otherwise. The OS and progression free survival (PFS) curves were estimated using the Kaplan–Meier method and their differences were analysed using the two‐sided log‐rank test. Tests used to evaluate statistical significance are detailed in the figure/table legends. All *p* values reported were two‐sided.

## 3 RESULTS

### 3.1 Clinical characteristics of patients

**FIGURE 1:**
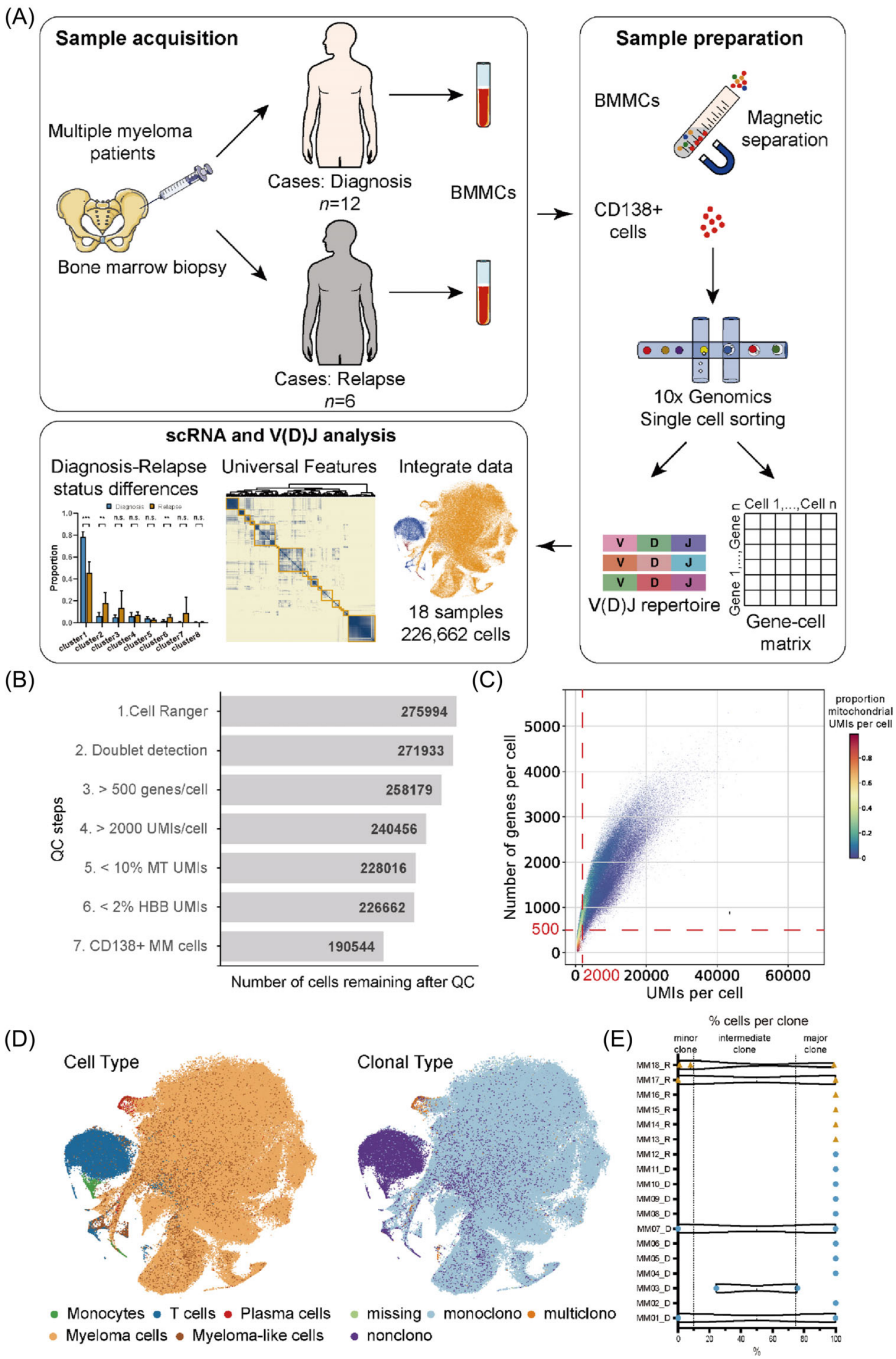
Study design, quality control and general characteristics. (A) Overview of study design, sample acquisition, preparation and analysis. We obtained the bone marrow mononuclear cells (BMMCs) from a Chinese multiple myeloma (MM) cohort (*n* = 12 for diagnosis and six for relapse), profiled CD138^+^ myeloma cells with scRNA‐seq and scVDJ‐seq to describe their transcriptional features and clonal diversity. (B) Cell counts over seven quality control (QC) steps. (C) Single‐cell QC metrics showed by scatter plot. Each cell was plotted according to the UMIs per cell, the number of genes per cell and the proportion mitochondrial UMIs per cell. QC thresholds were demarcated with dashed lines. UMIs, unique molecular identifiers. (D) t‐SNE visualization of all cells from 18 myeloma samples. Cells in the left graph were coloured according to cell types (monocytes, T cells, plasma cells, myeloma cells and myeloma‐like cells). Cells in the right graph were coloured according to clonal types (missing, nonclono, monoclono and multiclono). t‐SNE, t‐distributed stochastic neighbor embedding; missing, uncaught cells in scVDJ‐seq; nonclono, cells with missing scVDJ‐seq data. (E) Percentage of malignant cells per clone (triangle/dot) and patient (row). The clone frequency distribution for a given patient is represented with a violin plot

Concurrent single‐cell sequencing of tumour clonotypes with rearrangement of variable‐diversity‐joining regions and transcriptomes (scVDJ‐ and scRNA‐seq) was used to analyse BM CD138^+^ cells from 18 MM patients, including 12 NDMM subjects and 6 RRMM subjects, and an overview of experimental design is shown in Figure 1A. The median age of the 18 patients was 56 (range 37–70) years with 12 males and 6 females. Six, nine and three cases were with IgA, IgG and IgD monoclonal proteins, respectively. Cytogenetic analysis by FISH was a routine test at sample collection. Of the 18 patients, 14 harboured IgH translocation, including t(11;14) (*n* = 2), t(4;14) (*n* = 4) and translocation with an unknown partner (*n* = 8). Besides, 13q‐, 17p‐ and 1q21+ were found in three, two and six patients, respectively. All 12 NDMM patients received the uniform bortezomib /cyclophosphamide/dexamethasone (VCD) regimen as the first‐line therapy, while the six RRMM patients were resistant to multiple front‐line therapies (Table 1).

**TABLE 1:** Patients’ characteristics

Parameter	Total (*n* = 18)	Diagnosis (*n* = 12)	Relapse (*n* = 6)
Age (years) (median, range)	58 (34–74)	57 (46–74)	55 (37–70)
Male:female	12/6	8/4	4/2
Hb (g/L) (median, range)	91 (54–162)	97 (54–162)	89 (81–121)
Cr (μmol/L) (median, range)	79 (31–613)	86 (31–480)	70 (41–613)
LDH (U/L) (median, range)	241 (122–735)	317 (166–735)	205 (122–600)
β2‐MG (mg/L) (median, range)	5.05 (1.23–22.9)	8.32 (1.23/22.9)	3.59 (1.89–7.14)
M protein type, *n* (%)			
IgG	9 (50)	7 (58)	2 (33)
IgA	6 (33)	4 (33)	2 (33)
IgD	3 (17)	1 (8)	2 (33)
ISS stage, *n* (%)			
I	3 (17)	1 (8)	2 (33)
II	4 (22)	3 (25)	1 (17)
III	11 (61)	8 (67)	3 (50)
R‐ISS stage, *n* (%)			
I	2 (11)	0 (0)	2 (33)
II	9 (50)	7 (58)	2 (33)
III	7 (39)	5 (41)	2 (33)
FISH abnormalities, *n* (%)			
t(11;14)	2 (11)	2 (17)	0 (0)
t(4;14)	4 (22)	3 (25)	1 (17)
‐13/13q–	3 (17)	0 (0)	3 (50)
17p–	2 (11)	1 (8)	1 (17)
1q21+	6 (33)	2 (17)	4 (67)
Double‐hit	2 (11)	0 (0)	2 (33)
No adverse cytogenetics	8 (44)	6 (50)	2 (33)
Any high‐risk FISH	10 (55)	6 (50)	4 (67)
EMD, *n* (%)			
Bone adjacent	2 (11)	2 (17)	0 (0)
None‐bone	2 (11)	0 (0)	2 (33)
Best response, *n* (%)			
≧PR	14 (78)	11 (92)	3 (50)
<PR	4 (22)	1 (8)	3 (50)
OS (days) (median, range)	702 (39–758)	713 (304–758)	616 (39–732)

Abbreviations: EMD, extramedullary disease; ISS, International staging system; PR, partial response; R‐ISS, Revised international staging system; High‐risk, 17p‐, t(4;14), 1q21+; Double‐hit, > 1 high‐risk FISH abnormalities.

### 3.2 Identification of malignant cells and clonotypes in MM at single‐cell resolution

After quality control and filtering (Figure 1B), a total of 226 662 cells/transcriptomes were obtained from 18 samples, with a median of 1541 genes detected per cell (Table S1). We performed data integration and cell‐type annotation using the CCA algorithm based on gene expression profiling (GEP) (Figure 1D and Figures S2‐6). As expected, most of the CD138^+^ cells isolated by MACS were myeloma cells mixed with a few normal immunocytes, including T cells and monocytes (Figures S2‐6). Notably, the SDC1/CD138 gene product was identified as a weakly expressed in T cells in our study, which was consistent with previous findings that its expression was described in dendritic cells, activated T cells and macrophages.^26^, ^27^, ^28^, ^29^

As MM is a plasma cell neoplasm, myeloma cells undergo the same V(D)J recombination process and somatic hypermutation as B cells and often exhibit single‐cell clonal amplification.^30^ Next, we checked the malignant cell clonotypes through single‐cell V(D)J sequencing data. Clonotypes were distinguished by sequence difference in the CDR3 region of heavy and light chains through sequence alignment, and malignant cells with the same clonotype constituted a tumour clone. A total of 188 260 cells with complete BCR information were used to perform BCR analysis. In all patients, we detected a major clone with a dominated IGH‐IGL/IGK pair that existed in > 75% of malignant cells with defined clonotypes, consistent with the clonal expansion nature of this disease. Among the 18 samples, 13 samples consist of a single clone and the other five samples contain more than one clone (Figure 1E and Table S4). Notably, there was a patient at diagnosis (MM03_D) with a major MM clone (existed in 75.7% of the myeloma cells) and a subclone at intermediate frequencies (existed in 24.3% of the myeloma cells). We performed differential expression analysis between these two subclones and identified a list of genes statistically significant but subtle changed (log2FC < .5, Table S5), although the patient survived for only 1 year after diagnosis.

To further distinguish malignant cells from non‐malignant cells, we inferred large‐scale chromosomal CNVs based on transcriptomes^31^ and found that myeloma cells had a higher frequency of CNVs compared with immunocytes. Especially, the CNVs of chromosome 1q gain, 13q loss and 17p loss were observed in myeloma cells but not in immunocytes. This result was consistent with that detected by FISH (Figure S7 and Table 1).

### 3.3 Universal transcriptional features of myeloma cells

**FIGURE 2:**
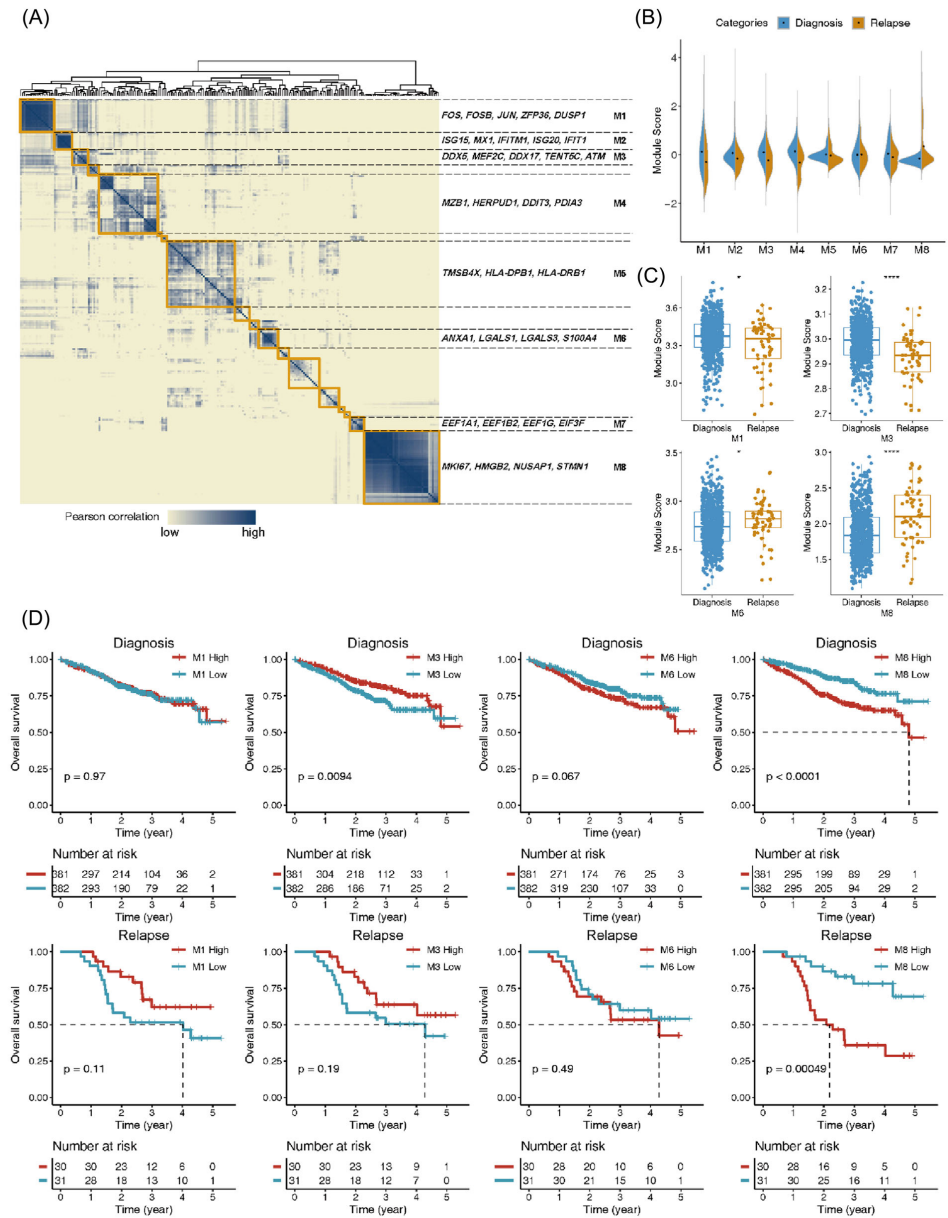
Universal features of multiple myeloma in different stages. (A) Heatmap showing 234 programs derived from cNMF analysis were correlated with MM, and eight highly correlated meta‐programs were highlighted. cNMF, consensus non‐negative matrix factorization. (B) Violin plots of the meta‐program score for each meta‐program. (C) Comparison of the module score between diagnosis subjects and relapse subjects from the MMRF CoMMpass study. ∗*p* < .05; ∗∗*p* < .01; ∗∗∗*p* < .001, ∗∗∗∗*p* < .0001. (D) Kaplan–Meier analysis of 843 subjects from the MMRF CoMMpass study. Subjects were divided into two groups according to the median value of the module scores, and *p* values were calculated by the log‐rank test

After filtering normal immunocytes, the remaining 190 513 cells were used to obtain the transcriptional signatures of myeloma cells (Table S1). cNMF algorithm^22^ was used to identify the intra‐tumour expression programs for each sample. According to previous reports, intra‐tumour expression programs in each sample reflecting universal features could be merged into meta‐programs.^32^, ^33^ By correlation clustering, we grouped 234 identified intra‐tumour expression programs into eight meta‐programs (Figure 2A, Figure S8 and Table S6), which covered diverse biological functions characterized by their top‐scoring genes, including stress response (M1; *FOS, FOSB, JUN, ZFP36* and *DUSP1*), type I interferon signalling pathway (M2; *ISG15, MX1, IFITM1, ISG20* and *IFIT1*), the transcriptional misregulation (M3; *DDX5, MEF2C, DDX17, TENT5C* and *ATM*), protein processing (M4; *MZB1; HERPUD1, DDIT3* and *PDIA3*), antigen processing and presentation (M5; *TMSB4X, HLA‐DPB1* and *HLADRB1*), leukocyte cell‐cell adhesion and migration (M6; *ANXA1, LGALS1, LGALS3* and *S100A4*), translation of mRNAs (M7; *EEEF1A1, EEF1B2, EEF1G* and *EIF3F*) and the cell cycle (M8; *MKI67, HMGB2, NUSAP1* and *STMN1*).

Then, we calculated module scores for myeloma cells based on the top 30 genes of each meta‐program and compared their differences between the NDMM and RRMM samples. The scores of meta‐programs 1–5 and 7 were significantly higher in NDMM samples than that in RRMM samples (all *p* < .001), while the scores of meta‐programs 6 and 8 were higher in RRMM samples (both *p* < .001) (Figure 2B and Figure S9). To validate these results, we compared the meta‐program scores between NDMM (*n* = 763) and RRMM (*n* = 80) samples with bulk RNA‐seq data from the MMRF CoMMpass study and observed similar trends on meta‐programs 1, 3, 6 and 8, but not on other meta‐programs (Figure 2C and Figure S10), which suggested that these four meta‐programs could be possible risk factors for MM relapse. Besides, we also found that the scores of meta‐programs 1, 3 and 8 differed significantly in a different series of matched diagnosis and relapse samples from 43 individuals (Figure S11). Next, we explored the correlation of meta‐programs scores with patient outcomes using Kaplan‐Meier analysis and the log‐rank test in the CoMMpass dataset. The results showed that the diagnosed or relapsed MM patients with higher scores of meta‐program 8 (M8) on myeloma cells had significantly poorer OS (Figure 2D; *p* < .0001 and *p* < .0005, respectively). The diagnosed MM patients with low scores of M3 or high scores of M6 and the relapsed MM patients with low scores of M1 or M3 were associated with relatively inferior OS (Figure 2D). Hierarchical clustering analysis revealed that relapsed individuals were enriched in groups 1 and 3, with a higher expression of cell cycle‐related genes (M8) and antigen processing‐related genes (M5), respectively (Figure S12).

### 3.4 Characterizing the cellular diversity at the single‐cell level

**FIGURE 3:**
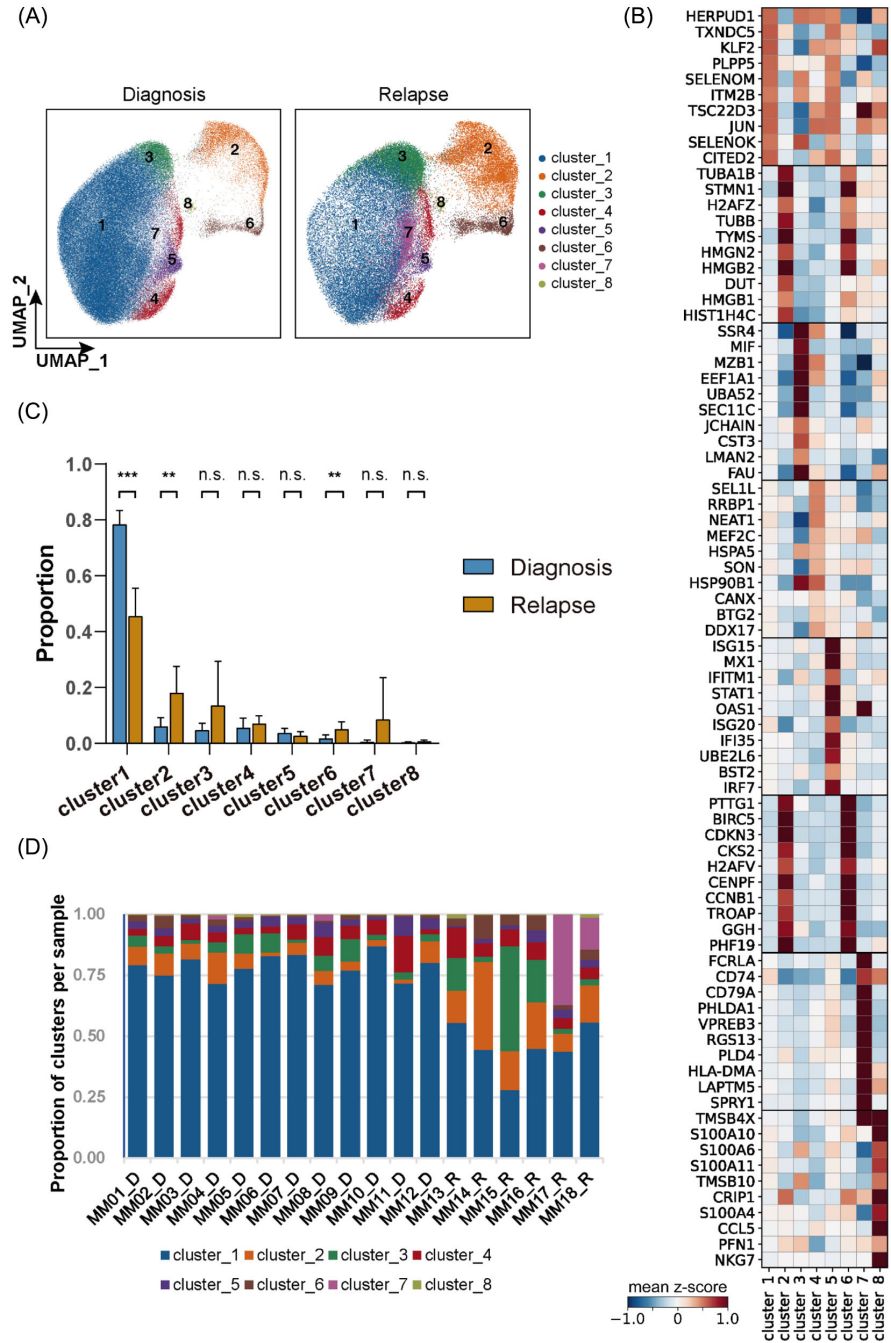
Cellular diversity of diagnosis and relapse MM samples. (A) UMAP visualization of all myeloma cells from 18 samples and cells were coloured according to clusters. UMAP, uniform manifold approximation and projection. (B) Heatmap showing signature genes of each cluster. (C) Comparison of cell proportions of each cluster between diagnosis and relapse MM samples. ∗*p* < .05; ∗∗*p* < .01; ∗∗∗*p* < .001, two‐sided unpaired Wilcoxon test. n.s, not significant. (D) Percentage bar plot showing the proportion of each cluster in each sample

Next, we combined the single‐cell transcriptomic data of all 18 individuals' myeloma cells using the CCA algorithm and visualized them using UMAP to investigate the diversity of cell population and transcriptional signatures between initial diagnosis and relapse (Figure 3A and Figures S13 and 14). We filtered out that human immunoglobulin heavy chain and light chain V segments (IGHV and IGLV/IGKV) for their high expression may influence the intrinsic transcriptional characteristics. Eight clusters with distinct transcriptional signatures were observed, and each cluster contained a different cell proportion for NDMM and RRMM samples (Figure 3A,B). Compared with the NDMM samples, the RRMM samples showed a significantly lower proportion of cells in cluster 1 and a higher percentage of cells in clusters 2 and 6 (Figure 3C). Notably, two RRMM samples (MM17_R and MM18_R) had a significantly higher proportion of cells in cluster 7 (37% and 13%) than others (Figure 3D).

### 3.5 Unique prognostic transcriptional factors identified by scRNA‐Seq

**FIGURE 4:**
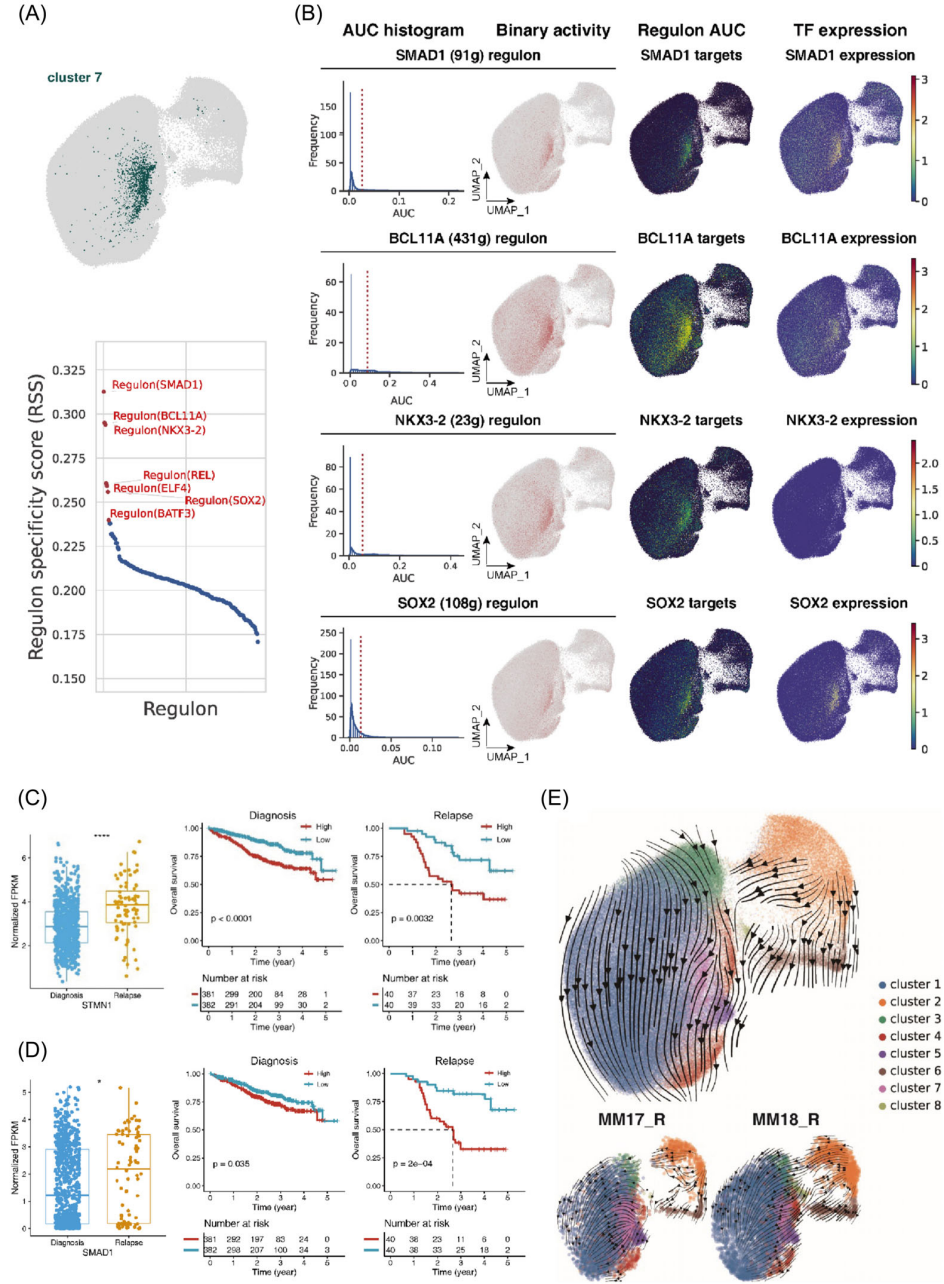
Transcription factor regulatory network and RNA velocity underlying myeloma cell phenotypes and states. (A) Regulon specificity score plot of cluster 7. The top regulons in cluster 7 were highlighted in red and labelled on the plot. The specificity score was shown on the *y* axis. (B) Comparison of transcription factor expression and regulon activity. In the first column: histogram of AUC values, together with the chosen threshold (orange dashed line). In the second column, the cells with AUC values over the threshold were shown in red and their regulon is considered active. In the third column, the actual AUC values were used to colour the cells. In the fourth column, the expression of the transcription factor itself was shown. AUC, area under the curve. (C) Box plots of the distribution of *STMN1* expression level for the samples from the CoMMpass data. ∗∗∗∗*p* < .0001, two‐sided unpaired Wilcoxon test. Kaplan–Meier analysis of initial diagnosis (top) or relapse (bottom) samples in the CoMMpass cohort. Two groups were divided according to the median value of *STMN1* expression level. The *p* values were calculated by the log‐rank test. (D) Box plots of the distribution of *SMAD1* expression level for the samples from the CoMMpass data. ∗∗∗∗*p* < .0001, two‐sided unpaired Wilcoxon test. Kaplan–Meier analysis of initial diagnosis (top) or relapse (bottom) samples in the CoMMpass cohort. Two groups were divided according to the median value of SMAD1 expression level. The *p* values were calculated by the log‐rank test. (E) RNA velocity plot of all 190 513 myeloma cells from 18 MM patients and myeloma cells from two individuals (MM17_R and MM18_R). Each dot represents a cell, and arrows indicate the directions of RNA velocity

We applied the single‐cell regulatory network inference and clustering (*SCENIC*) to analyse the clusters which had increased cell number exclusively in relapse samples. Since cells in cluster 7 were exclusively over‐presented in two RRMM samples (MM17_R and MM18_R), we performed the *SCENIC* analysis and found that four transcription factors (i.e. *SMAD1*, *BCL11A, NKX3‐2* and *SOX2*) together with their target genes were significantly up‐regulated in cluster 7 (Figure 4A,B and Table S8). Since *BCL11A* and *SOX2* were known as stem cell‐related transcription factors.^34^, ^35^ lineage scores of split clusters based on the expression signatures of different haematopoietic lineages were calculated (i.e. MMP, CLP, CMP, MEP, B‐cell and plasma)^36^ (Figure S15), and we found the expression signatures of MMP and B cells were highly represented in cluster 7, consistent with the finding of SCENIC analysis. Moreover, we found that high expression of *SMAD1* was associated with relapse risk and inferior OS, especially in relapsed patients (Figure 4D and Figure S16). Meanwhile, we also performed this analysis in cluster 1, which had a significantly lower proportion of cells in relapse samples, and found that the transcription factors (i.e. *ATF3, CREB3, ETV6* and *XBP1*) were specifically expressed (Figure S17). *ATF3* and *CREB3* bind to the cAMP‐response element and regulate cell proliferation.^37^, ^38^, ^39^ *ETV6* is required for haematopoiesis and maintenance of the developing vascular network,^40^, ^41^ and *XBP1* is a classical transcriptional regulator of plasma cell lineage.^42^ Taken together, it indicated that MM cells in cluster 1 retain plasma cell identity with proliferation status, which was in concordance with a recent study.^36^

As the cell proportions of clusters 2 and 6 were increased significantly in RRMM samples, we explored these clusters' signatures and found the proliferation and proteasome‐related genes were differentially expressed (Figure S18). Noteworthy, the lineage score analysis exhibited the expression signatures of immature progenitors (e.g. MMP and CLP) were also highly expressed in clusters 2 and 6 (Figure S10). Subsequently, we investigated the relevance of these gene expressions with MM prognosis using bulk RNA‐seq data from the MMRF CoMMpass study and found that increased levels of these gene expressions, such as *STMN1, TUBA1B, TUBB, TYMS* and *HMGN2* ranked the top five genes, were significantly associated with relapse risk and dismal OS (Figure 4C, Figure S19 and Table S7). To identify the overlap signature genes between our study and previous reports, an integrated comparative analysis was performed. Several sets of overlap genes enriched with pathways of oxidative phosphorylation and glycolysis/gluconeogenesis were found (Table S9 and Figure S20).

To explore the directional flow in cellular trajectories, we then performed RNA velocity analysis on all myeloma cells of the combined dataset through scVelo and validated the result on cells from single samples (Figure 4E). We noted that the velocity arrows pointed from clusters 2/6/7 to cluster 1 in the combined and individual datasets (MM17_R and MM18_R), suggesting that cells of clusters 2/6 and then cluster 7 may be in the earlier stages of malignant development than other cells in myeloma.

### 3.6 Functional validation of the genes identified

**FIGURE 5:**
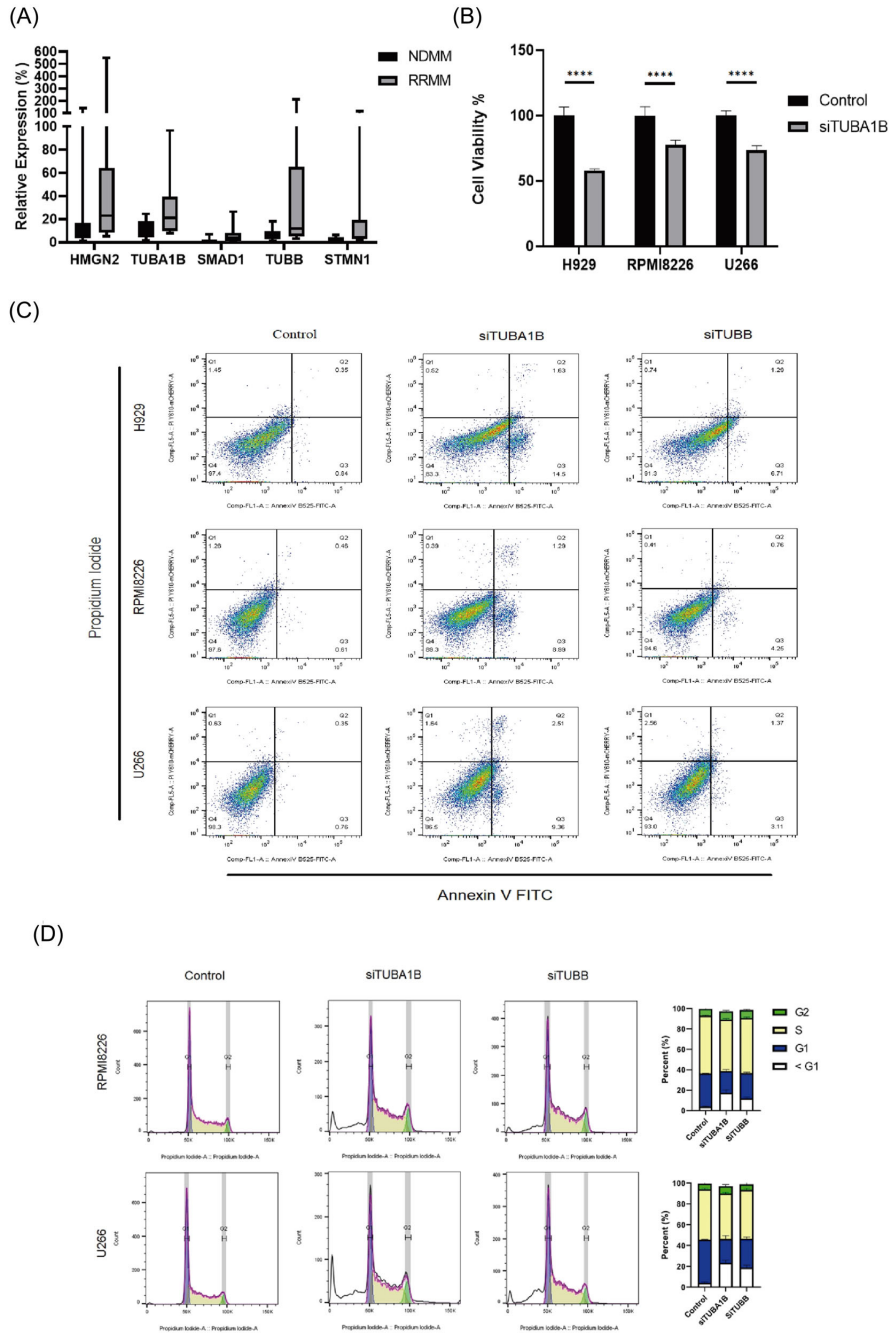
Functional validation of the genes identified by scRNA seq. (A) The expression levels of the five genes compared with the internal reference gene (β‐actin) were measured through qPCR in NDMM (*n* = 13) and RRMM (*n* = 12) samples. (B) The cell viability of three myeloma cell lines relative to the negative control after knocking down *TUBA1B*. (C) The apoptosis of three myeloma cell lines after knocking‐down of *TUBA1B* and *TUBB*. (D) The cell cycle measurement of U266 and RPMI8226 cell lines after knocking down of *TUBA1B* and *TUBB*

The representative genes identified by scRNA‐seq were candidates for further validation of their expression level between NDMM and RRMM patients based on RT‐qPCR assay, including *HMGN2, TUBA1B, SMAD1*, *TUBB* and *STMN1*. The median expression levels of these genes in RRMM patients were significantly higher than those in NDMM patients, which is shown in Figure 5A. And then, we focus on the *TUBA1B* and *TUBB* gene function analysis by knocked‐down in NCI‐H929, RPMI8226 and U266 myeloma cell lines. After knocking down *TUBA1B* gene, cell viability was significantly decreased in myeloma cells (Figure 5B), indicating that *TUBA1B* gene may impact on growth and proliferation of myeloma cell. Similarly, we observed that *TUBA1B* and *TUBB* regulate the levels and activities of a multitude of myeloma cells working on cell cycle and apoptosis in MM (Figure 5C,D).

## 4 DISCUSSION

Despite the advance in development with novel agents, myeloma relapse remains inevitable.^43^ Selective pressures contribute to genomic instability, clonal evolution, drug resistance^44^ and increased tumour burden. Understanding the clonal composition of MM helps guide therapeutic decision making by identifying combinations that can effectively target multiple subclones, therefore, single‐cell sequencing is a new methodology to dissect the unique cell type, the entire spectrum and clonal diversity of the transcriptome of individual cells. In this study, we applied this technology to systematically investigate MM in 18 patients at different stages of disease progression (newly diagnosis and relapse). We conducted scRNA and scVDJ sequencing for > 226K single cells, most of which were CD138^+^ malignant myeloma cells. We characterized the common features of myeloma cells and identified the cellular subpopulations, some of which were significantly over‐presented in the relapsed MM samples. We analysed differentially expressed genes (DEGs) and identified four potential transcriptional factors associated with disease progression and OS.

As MM is a plasma‐cell malignancy with clonal B‐cell origin, immunoglobulin heavy (IGH) and light chain V(D)J rearrangement sequences have been successfully exploited by next‐generation sequencing,^45^, ^46^ which is moving towards clinical implementation as a minimal residual disease (MRD) tracking.^47^, ^48^ We have applied the scVDJ analysis based on 10x Genomics to confirm the clonotype of tumour cells and found that 27.8% (5/18) MM samples possessed more than one clonotype. According to a previous report of bulk sequencing works, sub‐clones were rare and only found in 3.2% of MM patients by VDJ sequencing.^48^ Our work suggests that scVDJ sequencing may be a more sensitive method to illustrate the clonal diversity and could be a promising tool for MRD detection.

The transcriptional features of MM have been described by GEP analysis, and several gene expression signatures, including EMC92,^49^ UAMS70,^50^ UAMS80,^51^ IFM15,^52^ MRCIX6^53^ and GPI50,^54^ have been used for risk stratification in MM. Compared with GEP analysis, scRNA sequencing was able to more in‐depth interrogate heterogenetic clonal structures. In this study, all myeloma cells from 18 samples were analysed to characterize the transcriptional feature of MM, and a total of eight meta‐programs were identified to be correlated with this disease, in which the most significant one was meta‐program 8 covering the function of cell cycle consistent with the gene signature of IFM15, including 15 genes mainly involved in the cell cycle.^52^ Furthermore, meta‐program 1 covering stress response genes was also significantly correlated with MM, consistent with previous findings that pathways of endoplasmic reticulum stress^55^ and oxidative stress^56^ were extensively involved in myeloma. Few studies based on scRNA‐seq have revealed the disease‐progression or resistant signature of MM. One of the studies performed scRNA‐seq on samples from primary refractory patients enrolled in a trial of combination daratumumab, carfilzomib, lenalidomide and dexamethasone, and they identified a primary refractory signature and a broadly resistant signature, referred to as ‘PMID33619369 Module 1’ and ‘PMID33619369 Resistance signature 1’, respectively.^16^ Another study identified six coexisting transcriptional programs in single myeloma cells from RRMM patients, and among which program 5 (PMID34675390 P5 Cell cycle) was enriched with cell cycle genes and robustly expressed across patients.^36^ To identify the overlap genes between our study and previous reports, we performed an integrated comparative analysis between DEGs in each cluster (Table S9 and Figure S20).^16^, ^33^, ^36^, ^57^ We identified a slight overlap between our DEGs and other signature genes, which included a set of 14 genes increased expression in both clusters 2&6 and the ‘PMID33619369 Resistance signature 1’, indicating that both oxidative phosphorylation (i.e. *COX8A, COX6A1, NDUFB2, COX7B* and *NDUFS6*) and glycolysis/gluconeogenesis (i.e. *ENO1, TPI1* and *LDHA*) play roles in MM relapse. Meanwhile, a set of five gene intersections between clusters 2&6 and the ‘PMID34675390 P5 Cell cycle’ were identified as well, demonstrating proliferation state across MM cells. *PPIA* gene in a set of seven genes intersections among clusters 2&6, ‘PMID33619369 Resistance signature 1’ and ‘PMID33619369 Module 1’ has been defined as a potential target for resistance mechanism.^16^ Notably, most DEGs in clusters 2&6 and 7 were unique and novel results. Cell numbers in these clusters were exclusively higher in relapsed samples, suggesting the role of these clusters in MM progression and relapse.

Eight clusters of plasma cells were classified in this study, and the cell number in cluster 7 was exclusively higher in two relapsed samples (MM17_R and MM18_R). Moreover, we identified four genes, including *SMAD1, NKX3‐2, BCL11A* and *SOX2*, significantly expressed in this cluster. *SMAD1* was one of the essential molecules in the BMP/SMAD pathway critical for bone metabolism,^58^, ^59^ and *NKX3‐*2 was reported to relate with skeletal dysplasia.^60^ A recent study showed that *SMAD1* was highly expressed in RRMM cells and proved that *SMAD1* mediated MM drug resistance by regulating of NF‐κB1/TNFAIP8 and ID1‐p21/p27 axes,^61^ strongly supporting the analysis result from our scRNA‐seq data. *BCL11A* is essential for lymphoid development,^35^ and *SOX2* is a critical marker for stemness.^34^ How these four genes participate in the mechanism of myeloma progression needs to be further exploited. Besides, we found the clusters 2 and 6 were significantly over‐represented in RRMM samples with the top five up‐regulated signature genes (i.e. *STMN1, TUBA1B, TUBB, TYMS* and *HMGN2*), three out of which were found to be included in the identified GEP gene sets (i.e. *STMN1* in IFM15, *TUBB* in EMC92 and *TYMS* in GPI50). Moreover, *STMN1* and *TUBB* genes have been identified in similar expression programs associated with cell cycle and proliferation.^36^, ^62^ In the recent study based on scRNA‐seq, *STMN1* and *TYMS* were identified to be related to resistance pathways of myeloma cells.^16^ *STMN1*, a microtubule depolymerization protein, prevents assembly and promotes the disassembly of microtubules, thereby participating in the regulation of cell cycle, migration, apoptosis and other processes.^16^ *TUBA1B* and *TUBB* are tubulins as the components of the cytoskeleton and play a key role in cell mitosis and chromosome separation. High mobility group nucleosomal binding domain 2 (HMGN2) acts as a transcriptional modulator by binding to chromatin as a member of HMG superfamily. We subsequently validate the gene expression level of *HMGN2, TUBA1B, SMAD1, TUBB* and *STMN1* in NDMM and RRMM patients. In accordance with our analysis, the gene expression levels in RRMM patients were more highly correlated with mRNA expression than NDMM patients. And then, loss‐of‐function experiments with *TUBA1B* and *TUBB* knocked‐down in three myeloma cell lines, which illustrated that *TUBA1B* and *TUBB* regulated the levels and activities of a multitude of myeloma cells working on cell cycle, cell proliferation and apoptosis in MM. However, other's function needs to be further explored in the near future.

In summary, we have characterized the heterogeneous malignancy of MM at the single‐cell resolution and identified common and special transcriptomic and clonal features between cells from patients with diagnosed and relapsed MM. These findings shed light on the molecular and cellular complexity of MM and also potential molecular biomarkers for risk stratification and therapeutic options.

## CONFLICT OF INTERESTS

All the authors declare no conflict of interests.

## Supporting information



Supporting Information

Supporting Information
