# Supplementary material for: Single‐cell RNA‐seq reveals clonal diversity and prognostic genes of relapsed multiple myeloma
Source: Clin Transl Med. 2022 Mar 16;12(3):e757. doi: 10.1002/ctm2.757 (PMC8926895; doi:10.1002/ctm2.757)
Supplement: Supplementary file 1 — Supporting Information [file CTM2-12-e757-s001.pptx]

## Slide 1
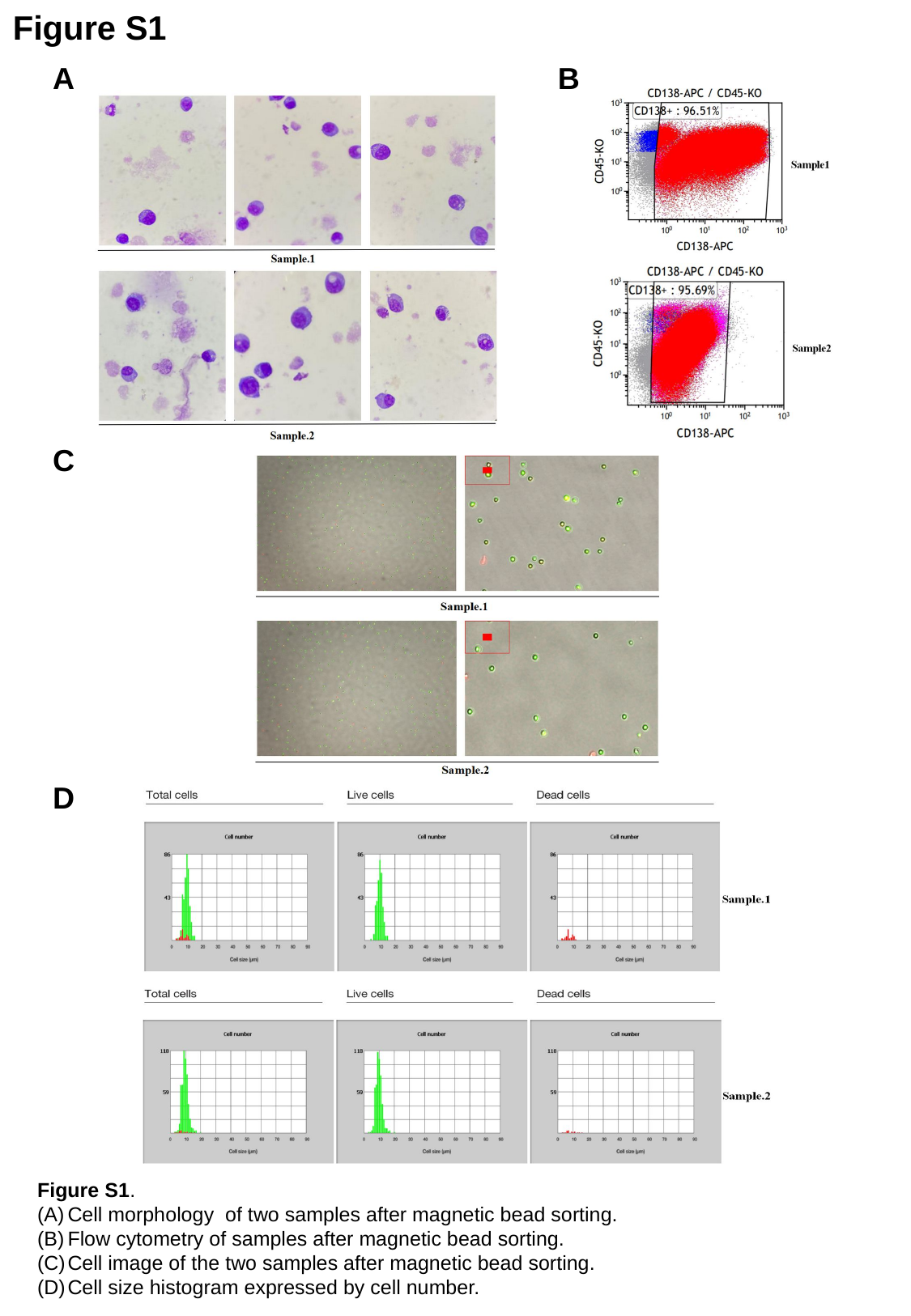

Figure S1
A
B
C
D
Figure S1.
Cell morphology of two samples after magnetic bead sorting.
Flow cytometry of samples after magnetic bead sorting.
Cell image of the two samples after magnetic bead sorting.
Cell size histogram expressed by cell number.

## Slide 2
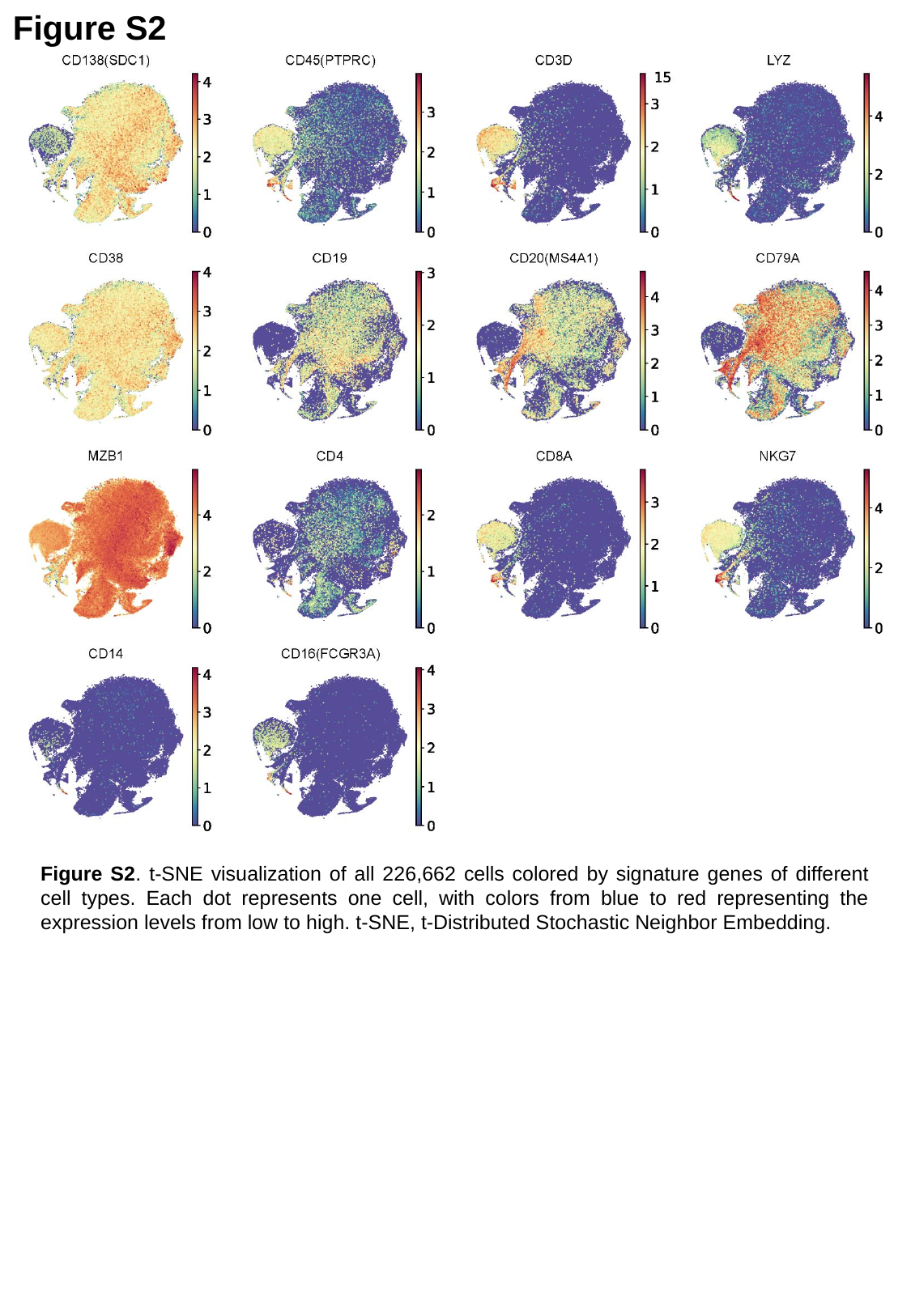

Figure S2
Figure S2. t-SNE visualization of all 226,662 cells colored by signature genes of different cell types. Each dot represents one cell, with colors from blue to red representing the expression levels from low to high. t-SNE, t-Distributed Stochastic Neighbor Embedding.

## Slide 3
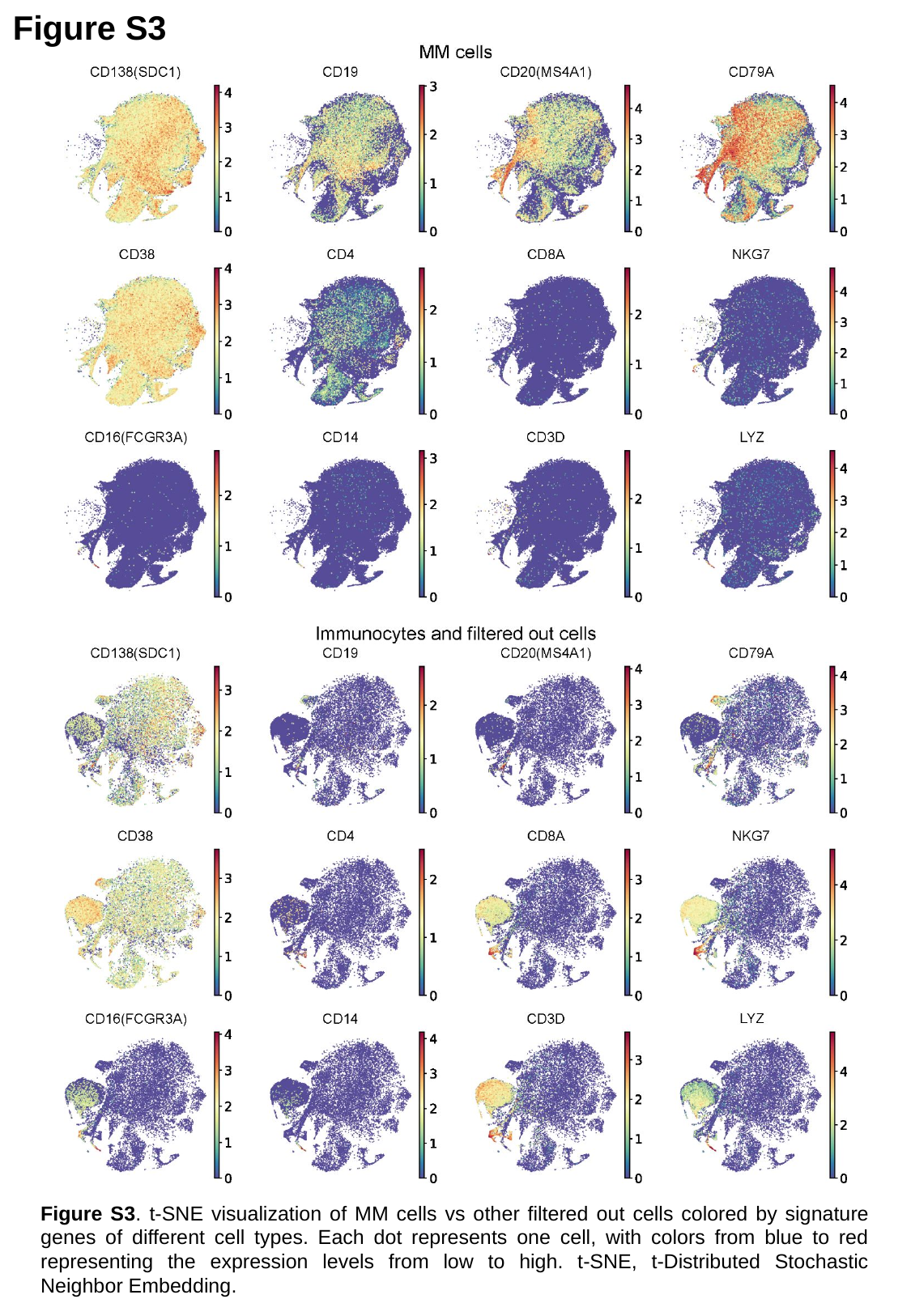

Figure S3
Figure S3. t-SNE visualization of MM cells vs other filtered out cells colored by signature genes of different cell types. Each dot represents one cell, with colors from blue to red representing the expression levels from low to high. t-SNE, t-Distributed Stochastic Neighbor Embedding.

## Slide 4
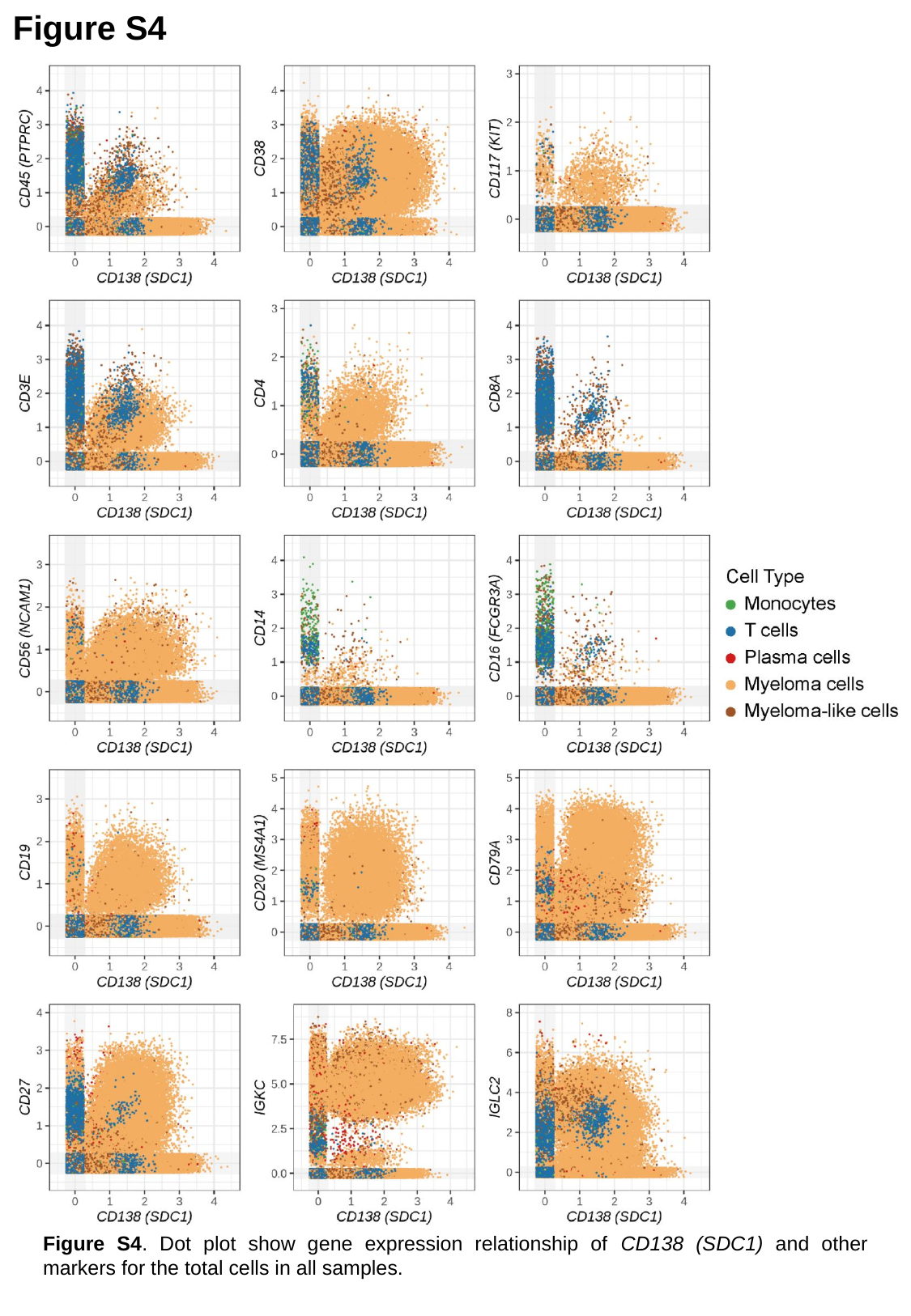

Figure S4
Figure S4. Dot plot show gene expression relationship of CD138 (SDC1) and other markers for the total cells in all samples.

## Slide 5
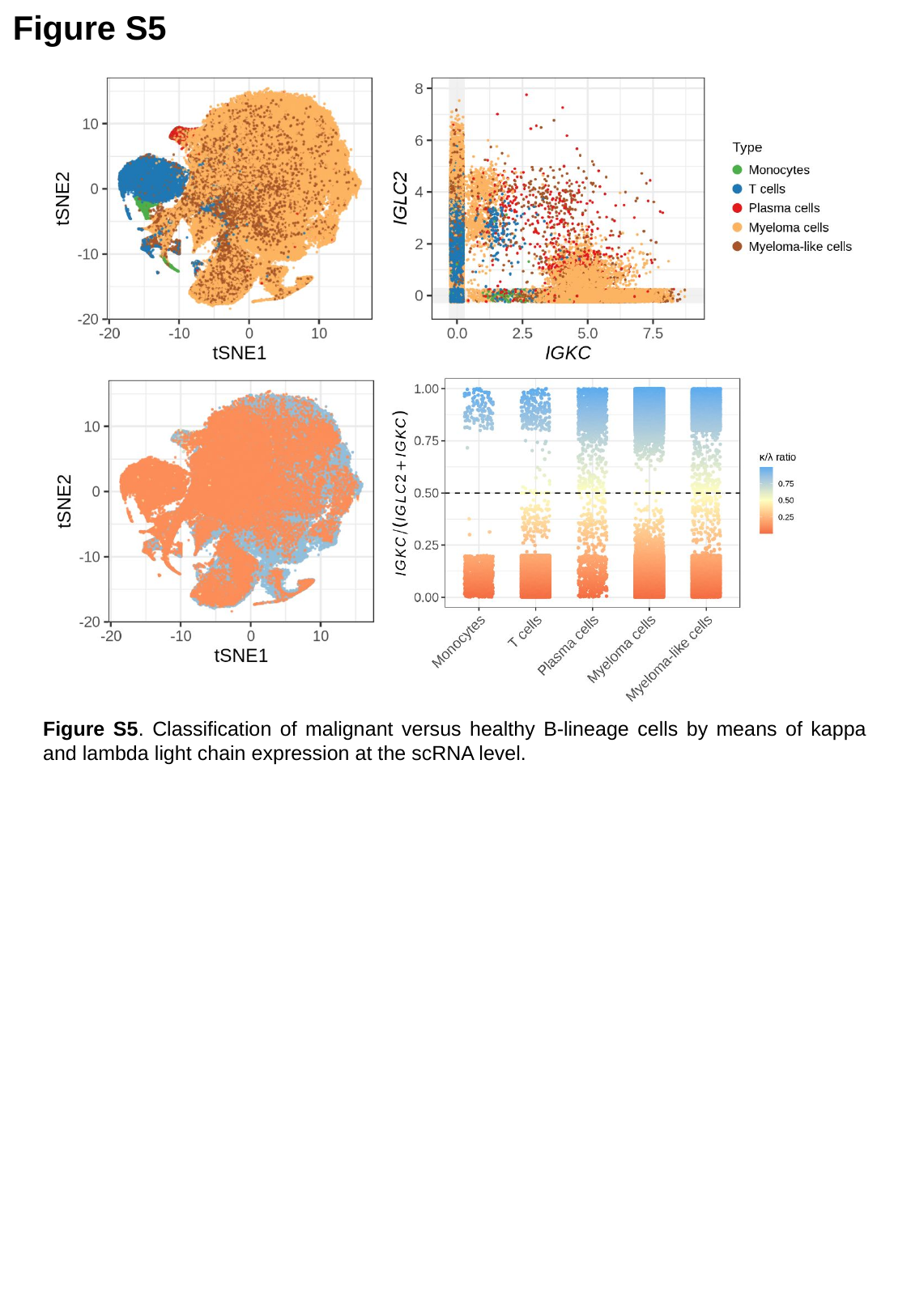

Figure S5
Figure S5. Classification of malignant versus healthy B-lineage cells by means of kappa and lambda light chain expression at the scRNA level.

## Slide 6
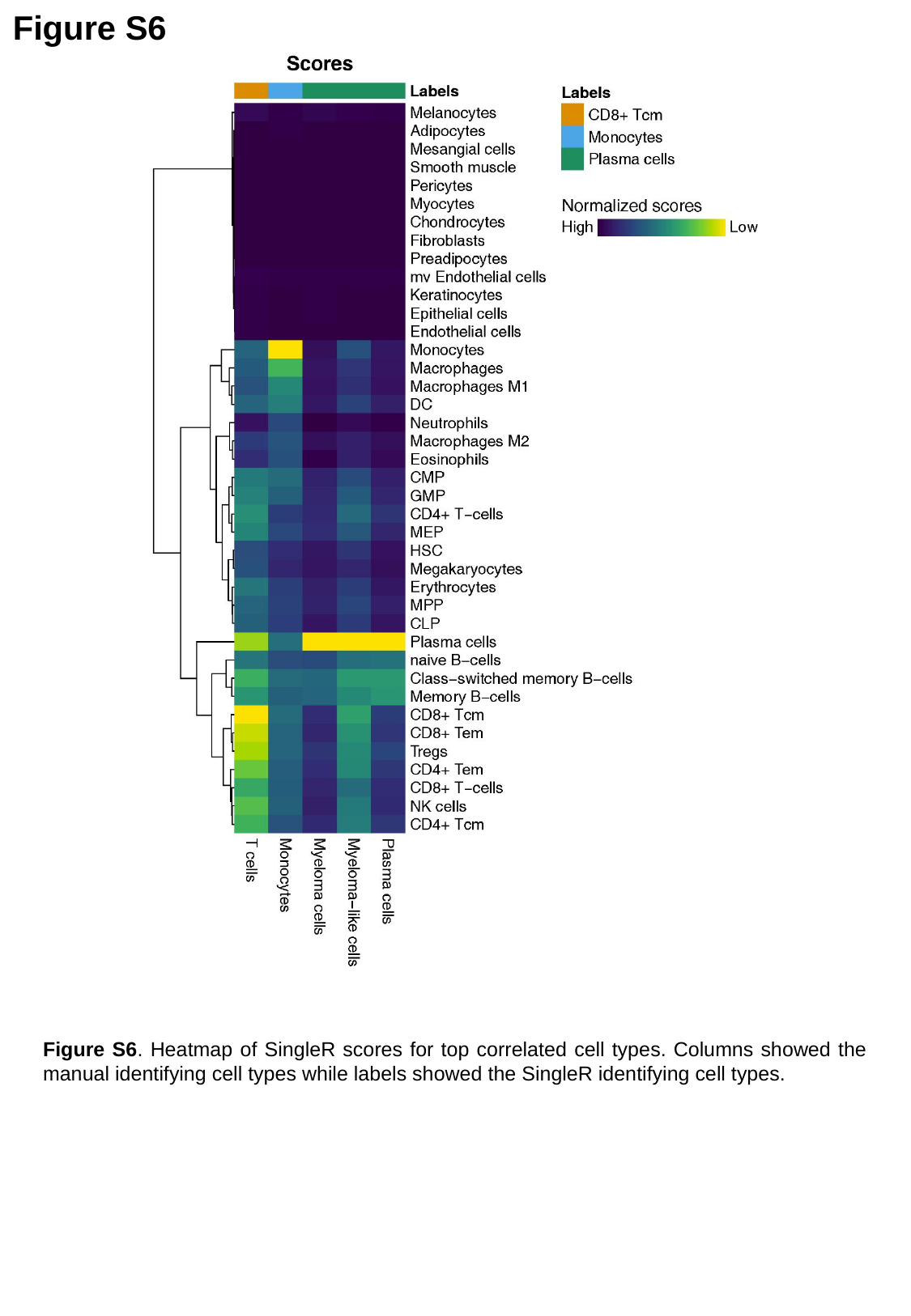

Figure S6
Figure S6. Heatmap of SingleR scores for top correlated cell types. Columns showed the manual identifying cell types while labels showed the SingleR identifying cell types.

## Slide 7
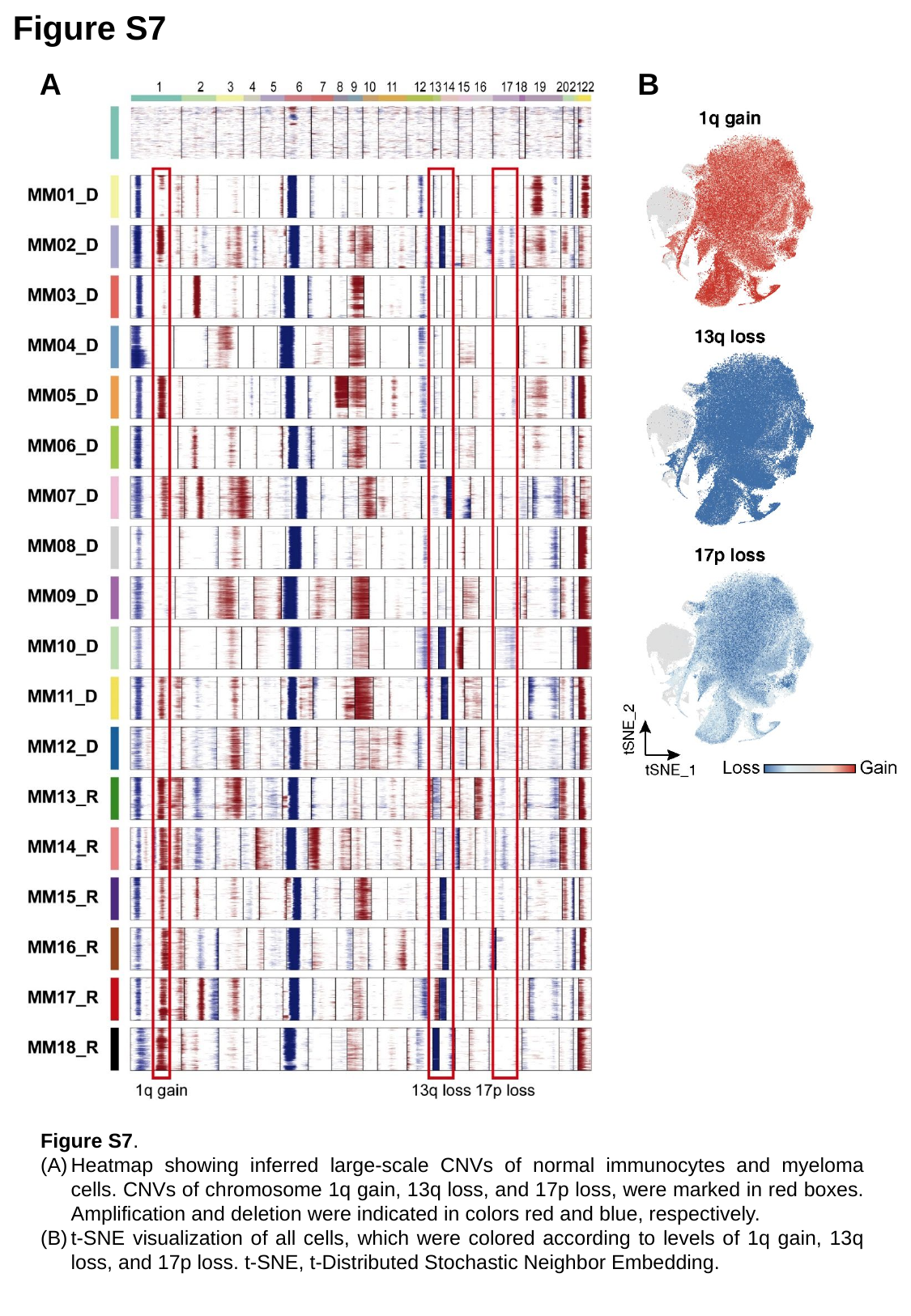

Figure S7
A
B
Figure S7.
Heatmap showing inferred large-scale CNVs of normal immunocytes and myeloma cells. CNVs of chromosome 1q gain, 13q loss, and 17p loss, were marked in red boxes. Amplification and deletion were indicated in colors red and blue, respectively.
t-SNE visualization of all cells, which were colored according to levels of 1q gain, 13q loss, and 17p loss. t-SNE, t-Distributed Stochastic Neighbor Embedding.

## Slide 8
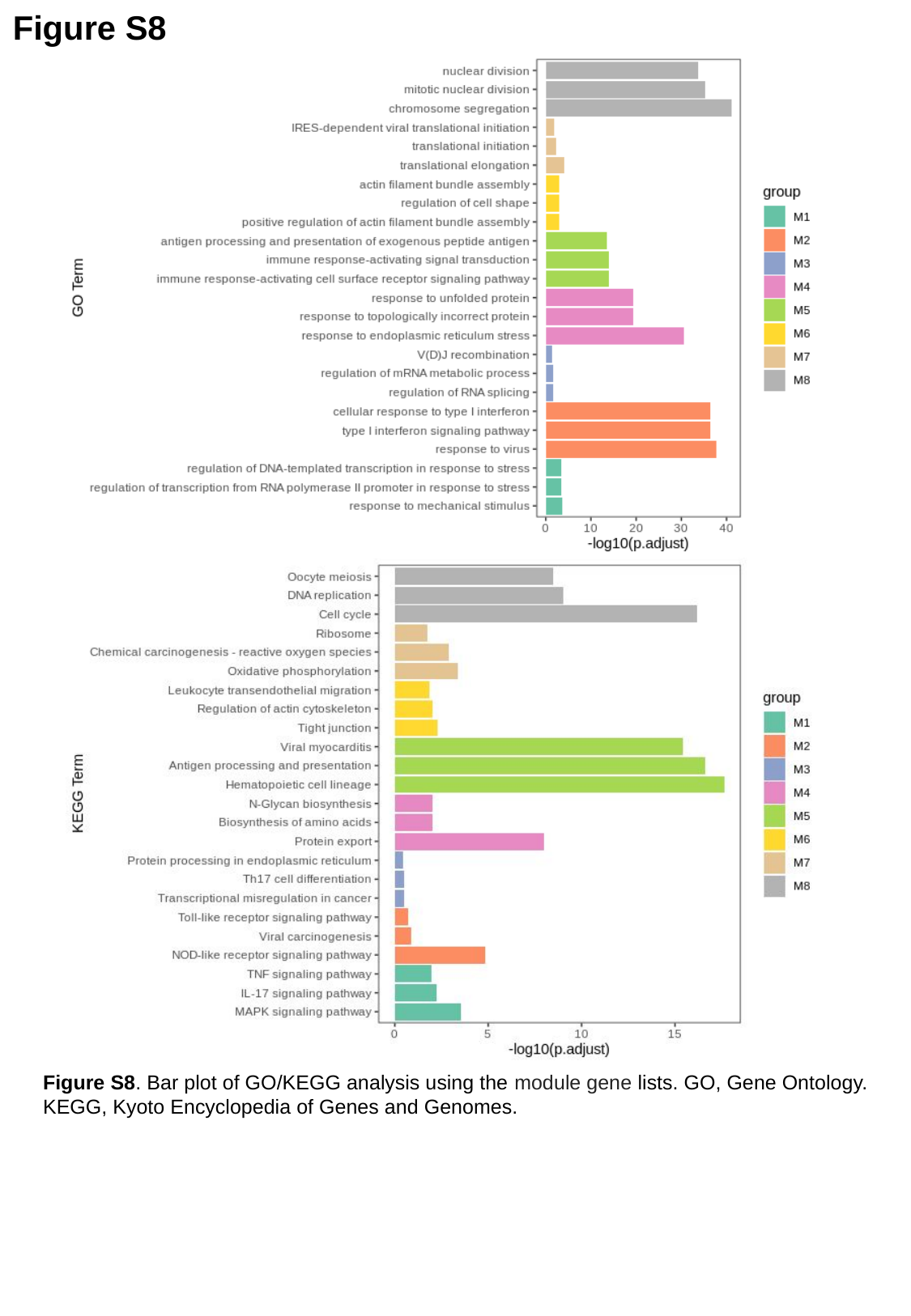

Figure S8
Figure S8. Bar plot of GO/KEGG analysis using the module gene lists. GO, Gene Ontology. KEGG, Kyoto Encyclopedia of Genes and Genomes.

## Slide 9
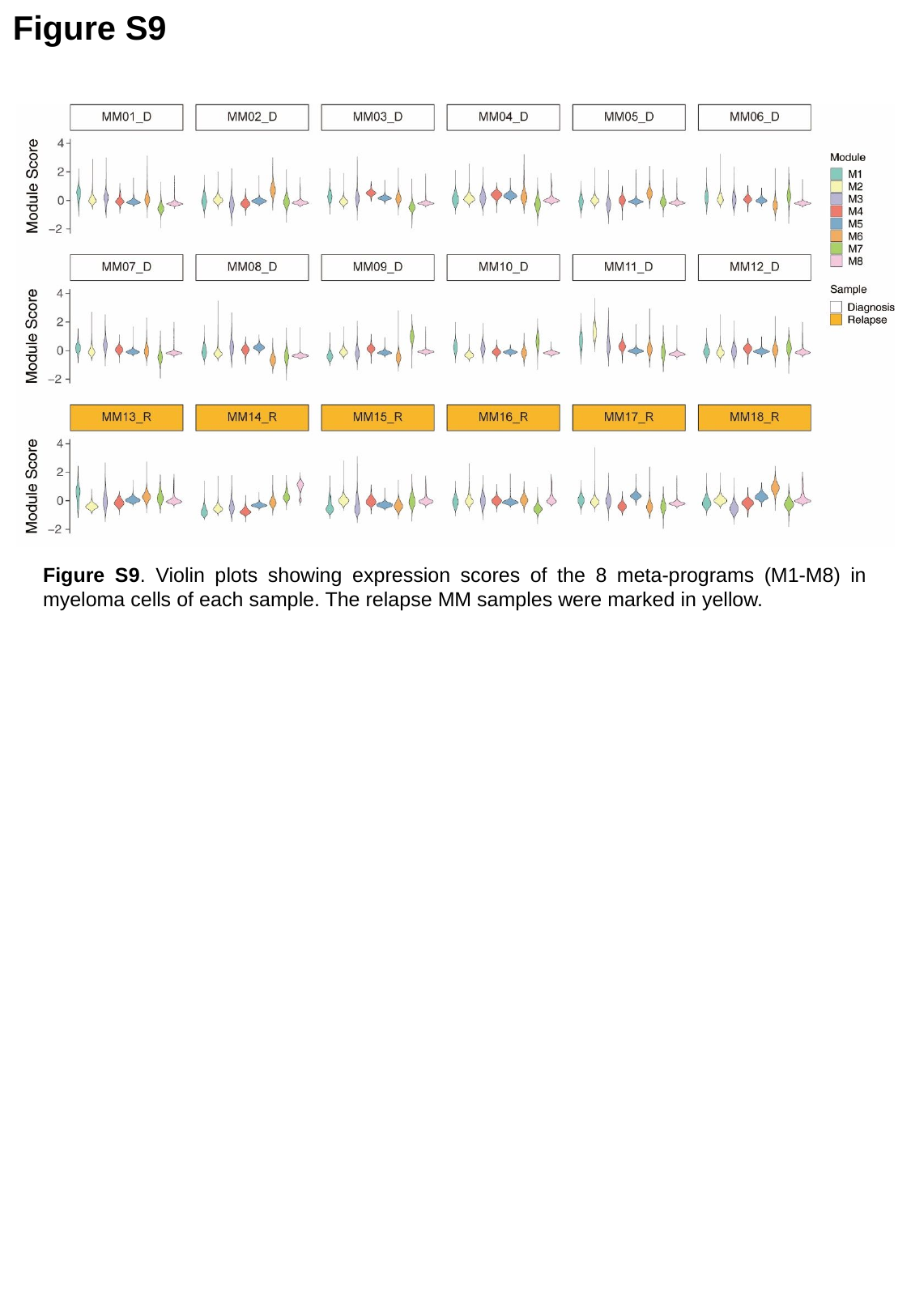

Figure S9
Figure S9. Violin plots showing expression scores of the 8 meta-programs (M1-M8) in myeloma cells of each sample. The relapse MM samples were marked in yellow.

## Slide 10
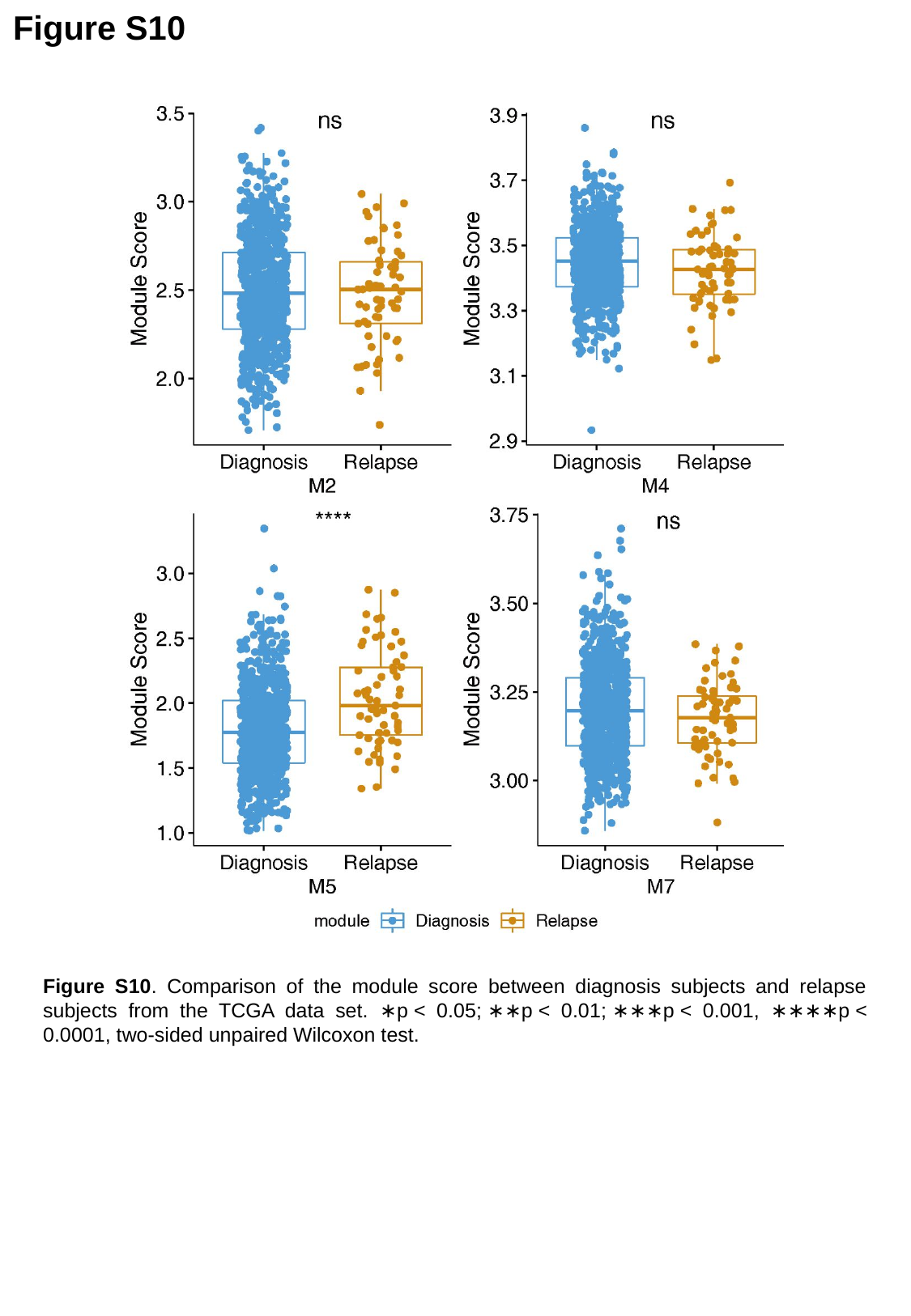

Figure S10
Figure S10. Comparison of the module score between diagnosis subjects and relapse subjects from the TCGA data set. ∗p < 0.05; ∗∗p < 0.01; ∗∗∗p < 0.001, ∗∗∗∗p < 0.0001, two-sided unpaired Wilcoxon test.

## Slide 11
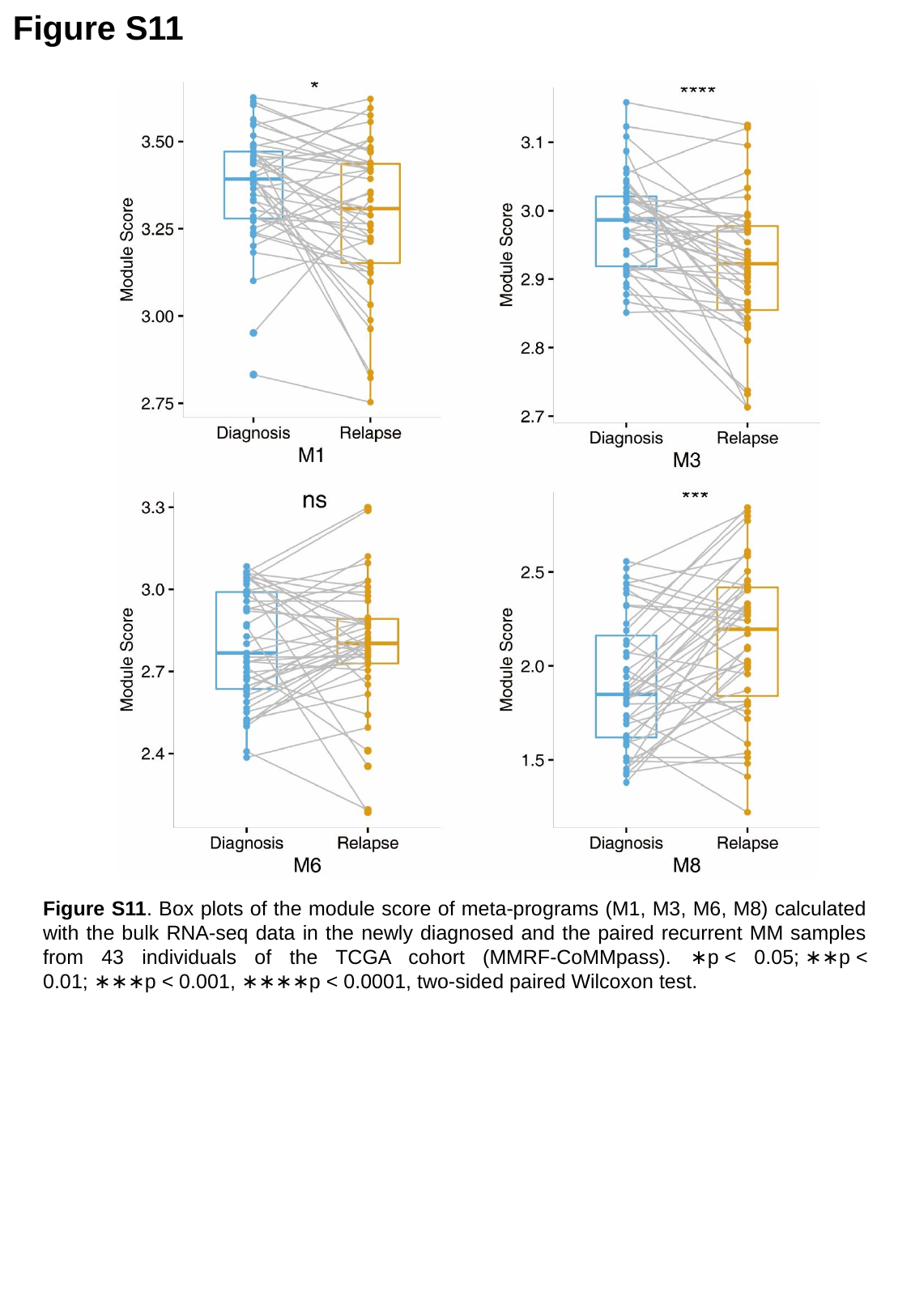

Figure S11
Figure S11. Box plots of the module score of meta-programs (M1, M3, M6, M8) calculated with the bulk RNA-seq data in the newly diagnosed and the paired recurrent MM samples from 43 individuals of the TCGA cohort (MMRF-CoMMpass). ∗p < 0.05; ∗∗p < 0.01; ∗∗∗p < 0.001, ∗∗∗∗p < 0.0001, two-sided paired Wilcoxon test.

## Slide 12
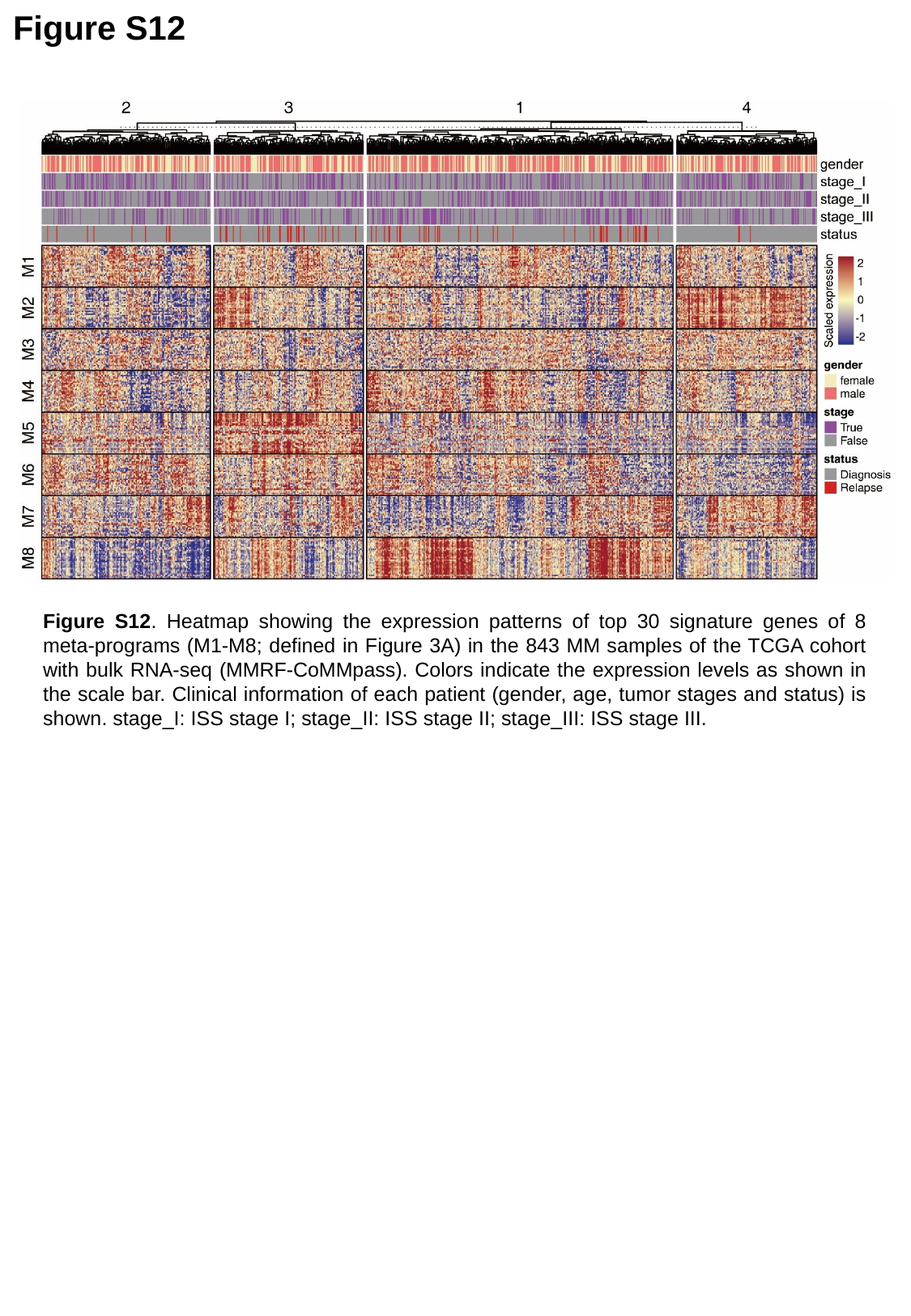

Figure S12
Figure S12. Heatmap showing the expression patterns of top 30 signature genes of 8 meta-programs (M1-M8; defined in Figure 3A) in the 843 MM samples of the TCGA cohort with bulk RNA-seq (MMRF-CoMMpass). Colors indicate the expression levels as shown in the scale bar. Clinical information of each patient (gender, age, tumor stages and status) is shown. stage_I: ISS stage I; stage_II: ISS stage II; stage_III: ISS stage III.

## Slide 13
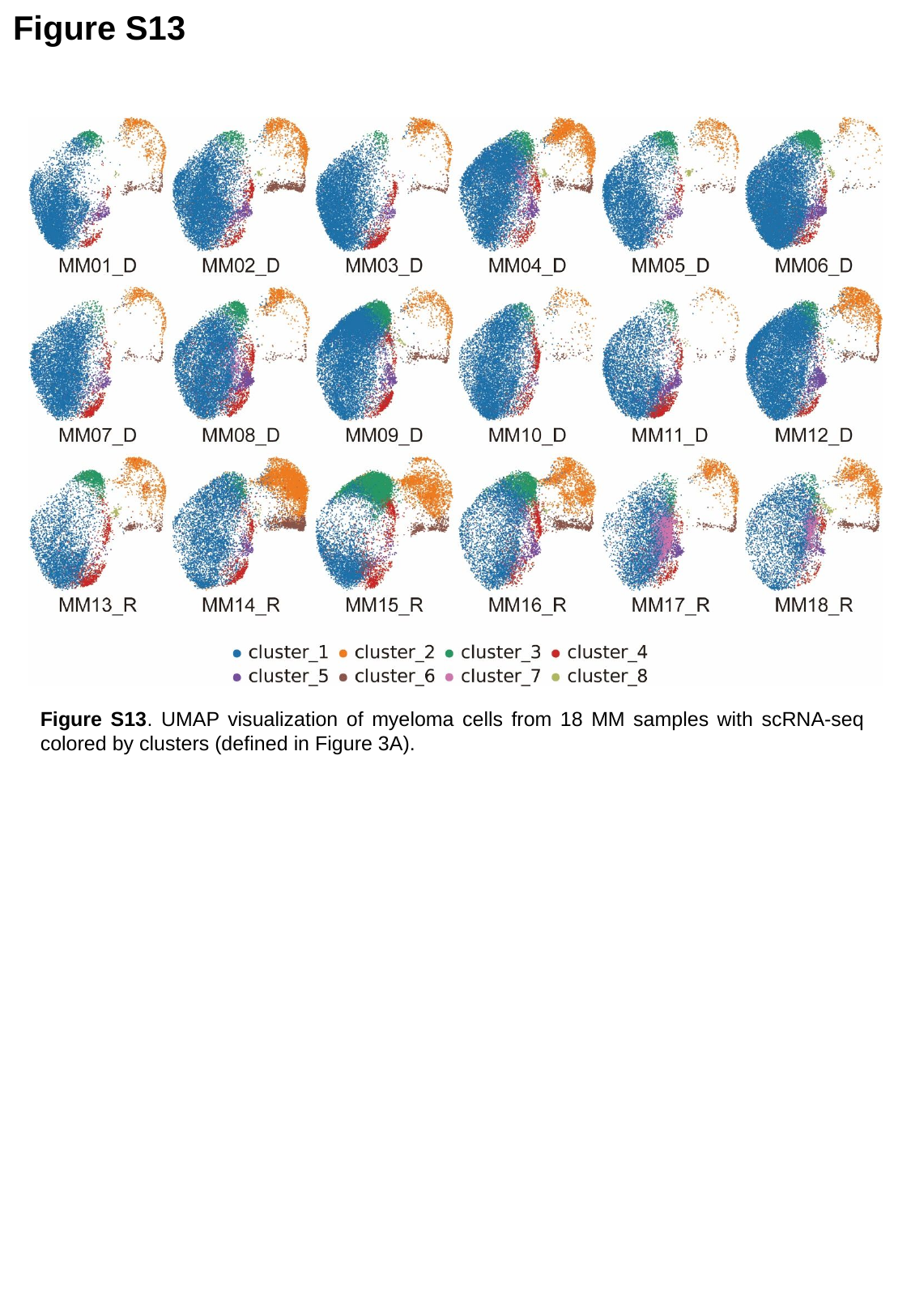

Figure S13
Figure S13. UMAP visualization of myeloma cells from 18 MM samples with scRNA-seq colored by clusters (defined in Figure 3A).

## Slide 14
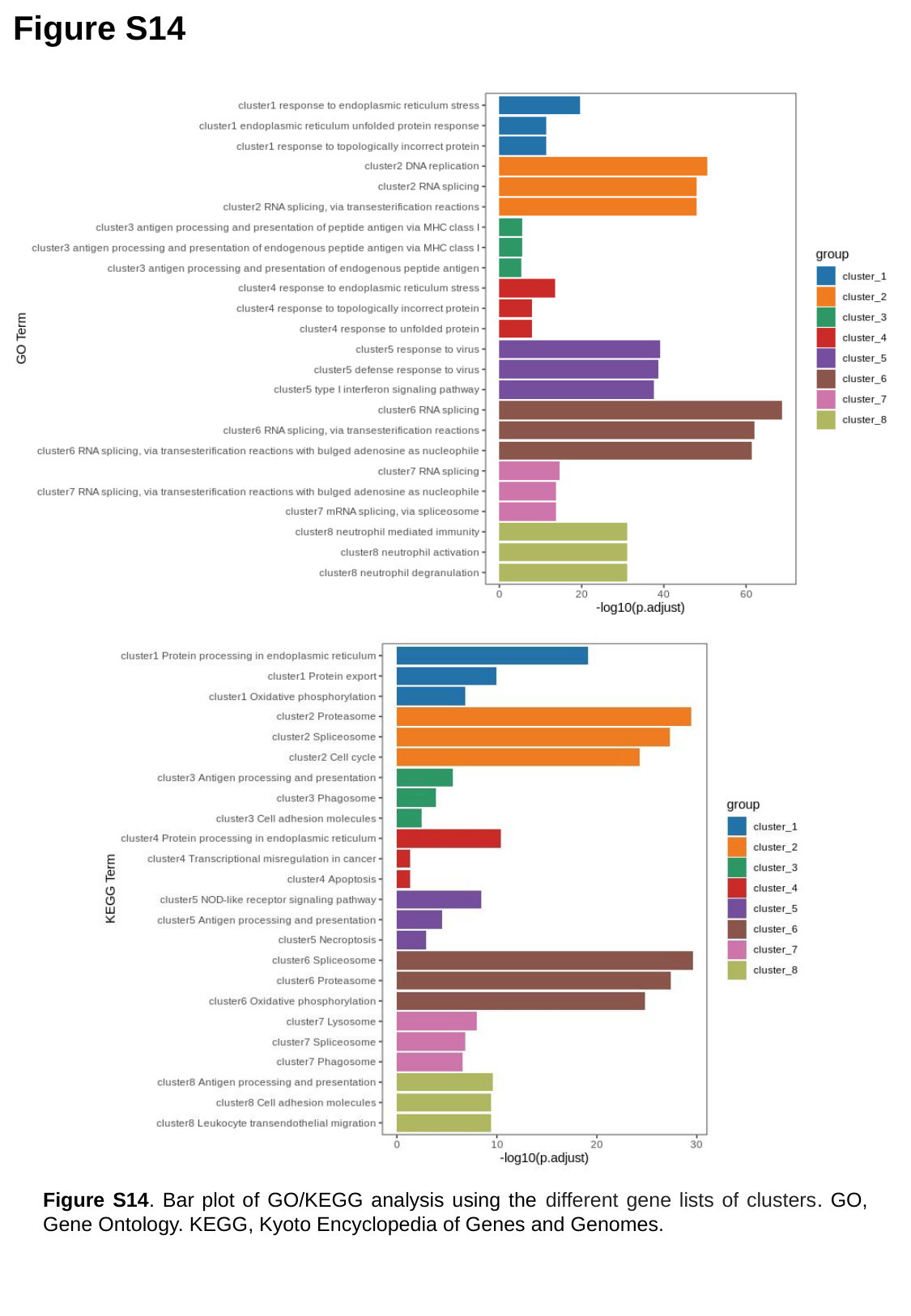

Figure S14
Figure S14. Bar plot of GO/KEGG analysis using the different gene lists of clusters. GO, Gene Ontology. KEGG, Kyoto Encyclopedia of Genes and Genomes.

## Slide 15
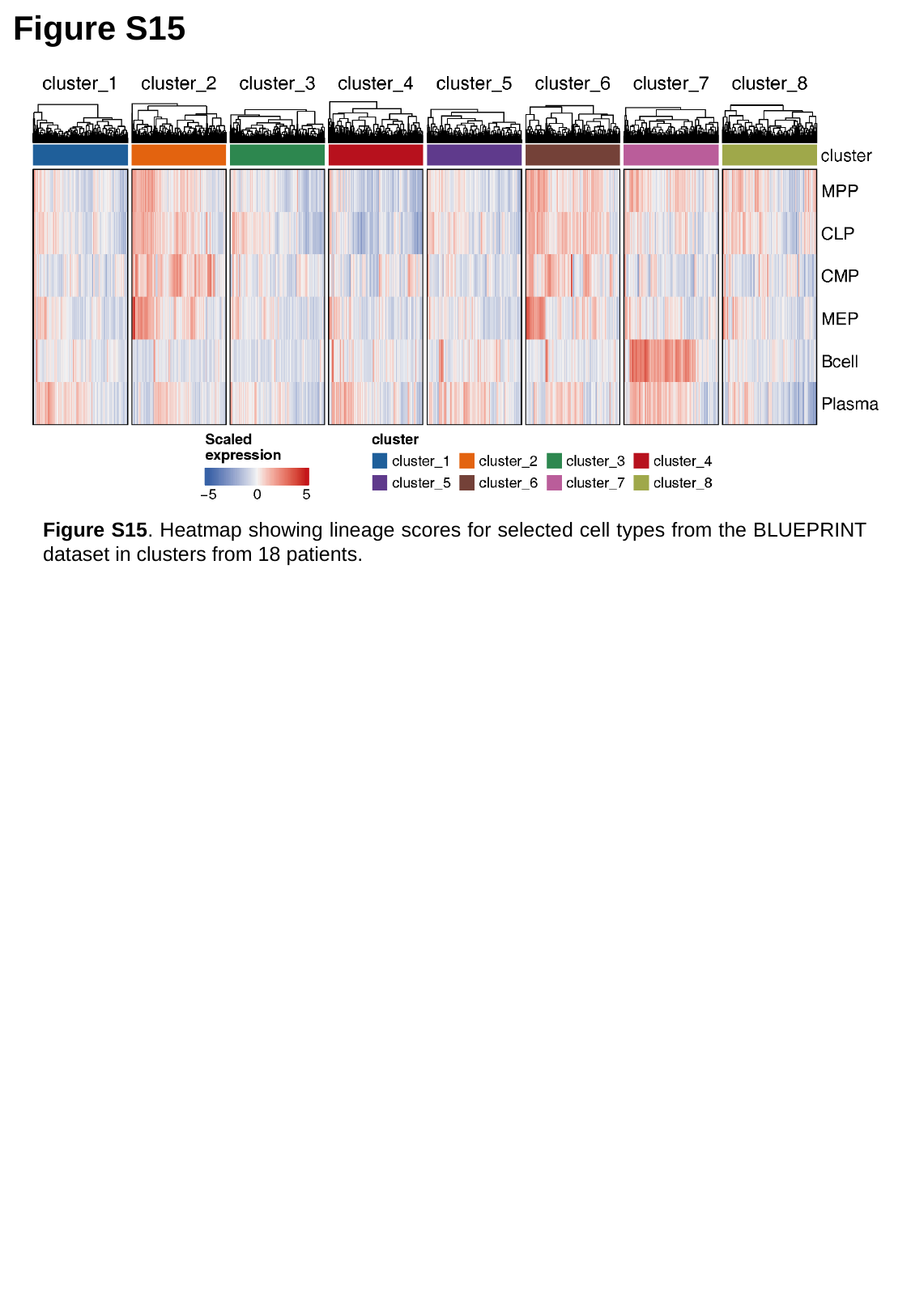

Figure S15
Figure S15. Heatmap showing lineage scores for selected cell types from the BLUEPRINT dataset in clusters from 18 patients.

## Slide 16
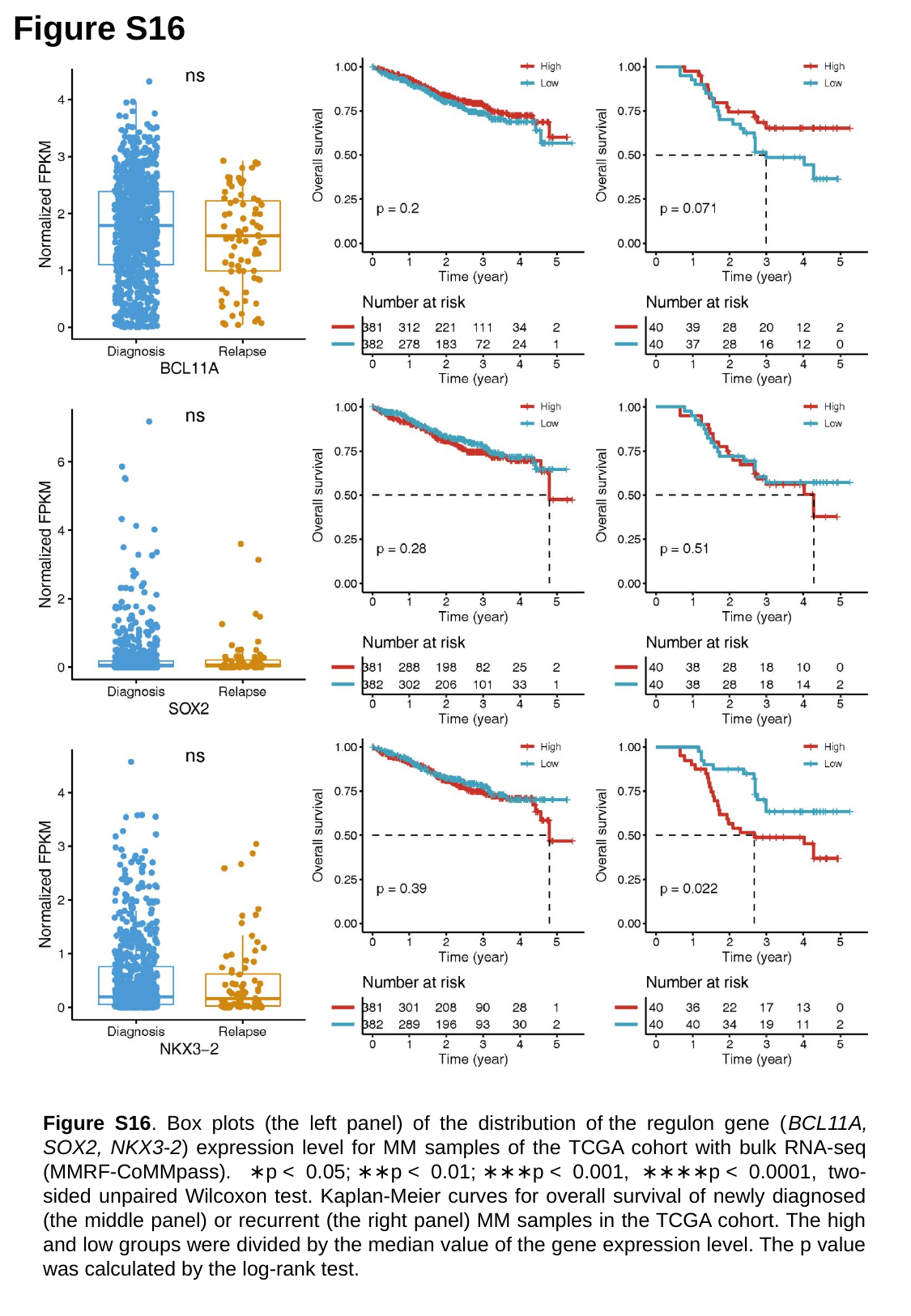

Figure S16
Figure S16. Box plots (the left panel) of the distribution of the regulon gene (BCL11A, SOX2, NKX3-2) expression level for MM samples of the TCGA cohort with bulk RNA-seq (MMRF-CoMMpass).  ∗p < 0.05; ∗∗p < 0.01; ∗∗∗p < 0.001, ∗∗∗∗p < 0.0001, two-sided unpaired Wilcoxon test. Kaplan-Meier curves for overall survival of newly diagnosed (the middle panel) or recurrent (the right panel) MM samples in the TCGA cohort. The high and low groups were divided by the median value of the gene expression level. The p value was calculated by the log-rank test.

## Slide 17
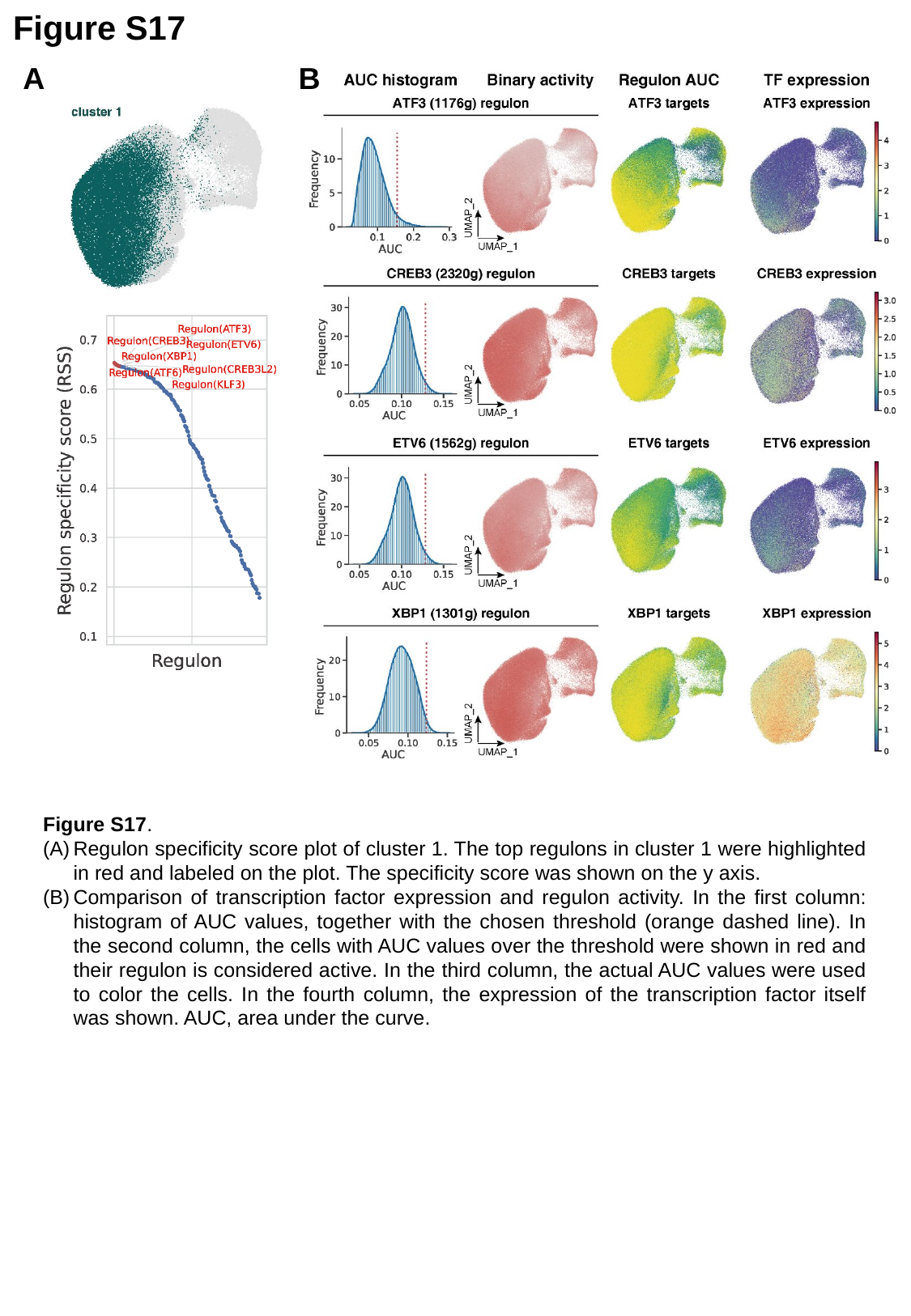

Figure S17
A
B
Figure S17.
Regulon specificity score plot of cluster 1. The top regulons in cluster 1 were highlighted in red and labeled on the plot. The specificity score was shown on the y axis.
Comparison of transcription factor expression and regulon activity. In the first column: histogram of AUC values, together with the chosen threshold (orange dashed line). In the second column, the cells with AUC values over the threshold were shown in red and their regulon is considered active. In the third column, the actual AUC values were used to color the cells. In the fourth column, the expression of the transcription factor itself was shown. AUC, area under the curve.

## Slide 18
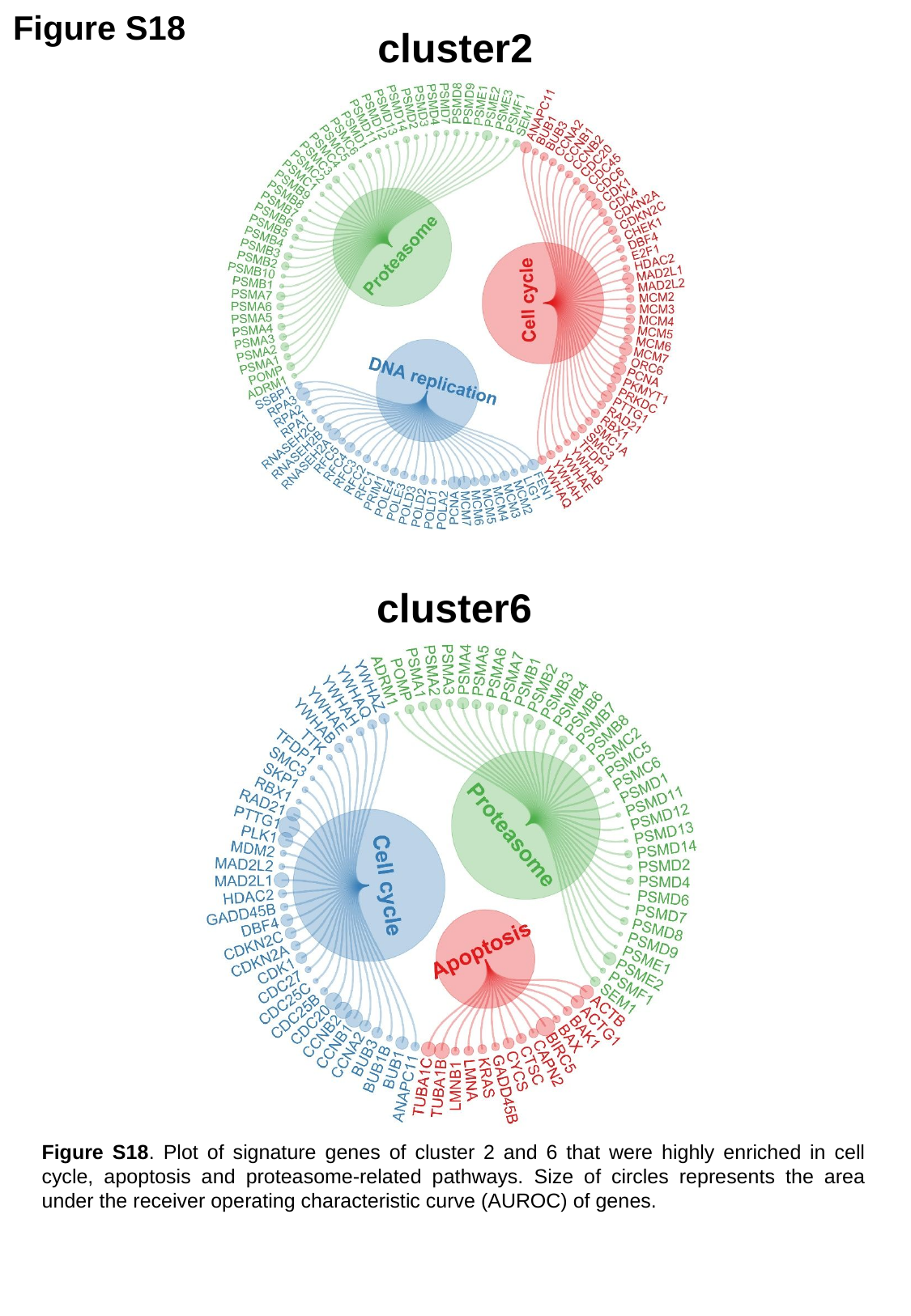

Figure S18
cluster2
cluster6
Figure S18. Plot of signature genes of cluster 2 and 6 that were highly enriched in cell cycle, apoptosis and proteasome-related pathways. Size of circles represents the area under the receiver operating characteristic curve (AUROC) of genes.

## Slide 19
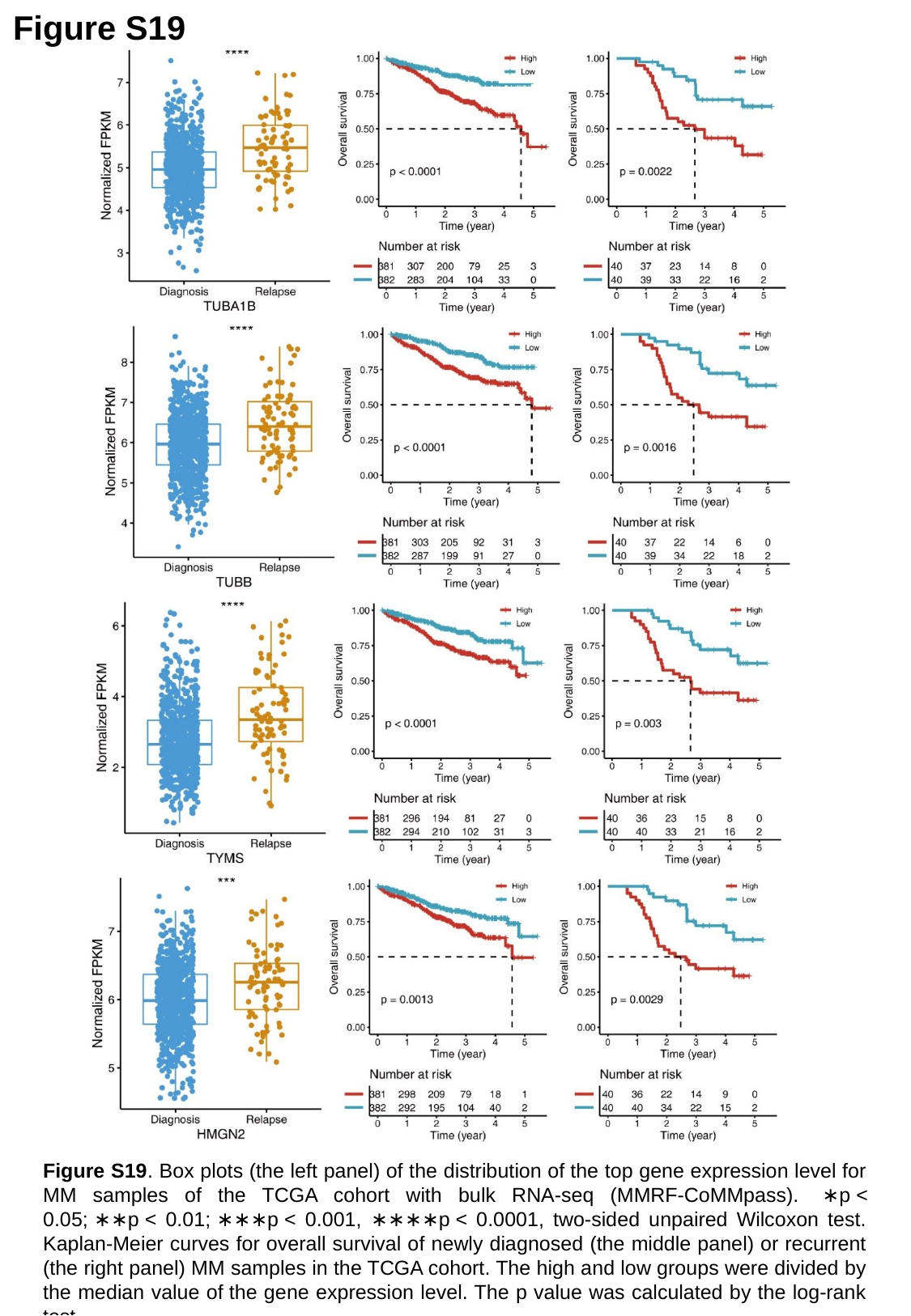

Figure S19
Figure S19. Box plots (the left panel) of the distribution of the top gene expression level for MM samples of the TCGA cohort with bulk RNA-seq (MMRF-CoMMpass).  ∗p < 0.05; ∗∗p < 0.01; ∗∗∗p < 0.001, ∗∗∗∗p < 0.0001, two-sided unpaired Wilcoxon test. Kaplan-Meier curves for overall survival of newly diagnosed (the middle panel) or recurrent (the right panel) MM samples in the TCGA cohort. The high and low groups were divided by the median value of the gene expression level. The p value was calculated by the log-rank test.

## Slide 20
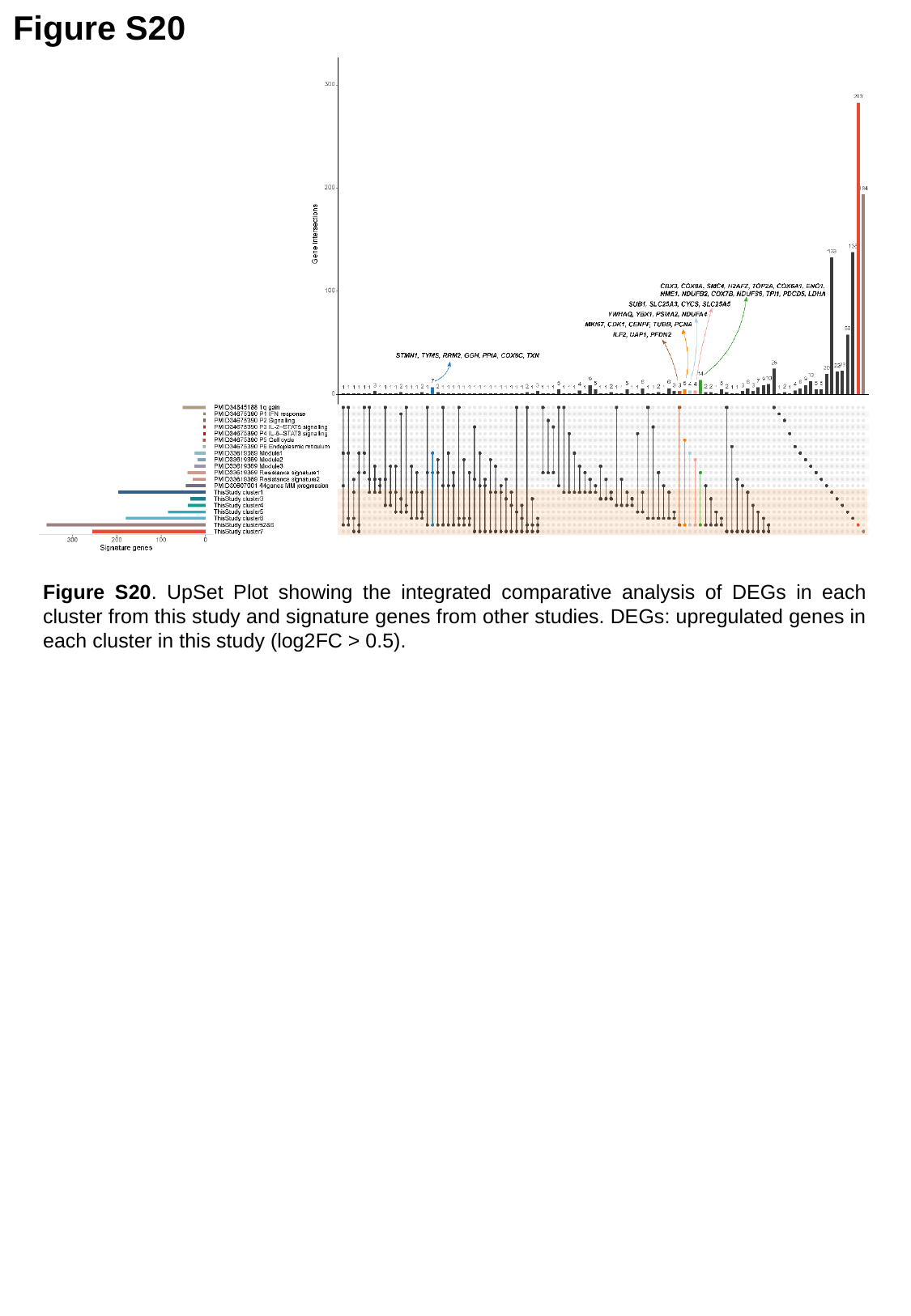

Figure S20
Figure S20. UpSet Plot showing the integrated comparative analysis of DEGs in each cluster from this study and signature genes from other studies. DEGs: upregulated genes in each cluster in this study (log2FC > 0.5).
